# Immune-related adverse events associated with nab-paclitaxel/paclitaxel combined with immune checkpoint inhibitors: a systematic review and network meta-analysis

**DOI:** 10.3389/fimmu.2023.1175809

**Published:** 2023-07-14

**Authors:** Wenjing Hao, Jun Zhang, Yunxia Wang, Boyu Fang, Shasha Jin, Jing Yuan, Weimin Cai

**Affiliations:** ^1^ Department of Clinical Pharmacy and Pharmacy Administration, School of Pharmacy, Fudan University, Shanghai, China; ^2^ School of Pharmacy, Minhang Hospital, Fudan University, Shanghai, China

**Keywords:** immune-related adverse events, systematic literature review, network meta-analysis, immune checkpoint inhibitors, nab-paclitaxel, paclitaxel

## Abstract

**Objective:**

The combination of nanoparticle albumin-bound paclitaxel (nab-PTX)/paclitaxel (PTX) with immune checkpoint inhibitors (ICIs) has demonstrated significant efficacy in cancer patients. However, the safety of these combination regimens remains conflicting in former researches. Therefore, in order to address this issue, we performed a systematic review and network meta-analysis (NMA) to evaluate and compare the safety profile.

**Methods:**

We performed a systematic review by searching randomized controlled trials (RCTs) from PubMed, EMBASE, Cochrane Library, ClinicalTrials.gov, and Web of Science up to August 15, 2022. The primary outcomes were all‐grade (grade 1‐5) and high‐grade (grade 3‐5) immune-related adverse events (irAEs). Secondary outcomes were all‐grade (grade 1‐5) and high‐grade (grade 3‐5) irAEs of subgroups of ICIs.

**Results:**

There were 22 RCTs included in the NMA, involving a total of 15 963 patients diagnosed with any type of cancer. ICIs+nab-PTX was associated with a noticeably decreased risk of grade 3-5 pneumonitis (odds ratio [OR]=0.28, 95% credible interval [CrI]: 0.09,0.90) compared to ICI monotherapy; ICIs+PTX showed a lower risk of grade 1-5 hyperthyroidism (OR=0.46, 95% CrI: 0.22-0.96) and grade 1-5 hypothyroidism (OR=0.49, 95% CrI: 0.26-0.93) than ICIs. Compared with PD-1, PD-1+PTX was associated with a statistically significantly lower risk of grade 1-5 pneumonitis (OR=0.32, 95% CrI: 0.11-0.92). PD-L1 resulted in a noticeably lower risk of grade 1-5 hypothyroidism (OR=0.34, 95% CrI: 0.12-1.00) than PD-L1+PTX. Nearly all treatment regimens containing ICIs demonstrated significantly higher risks of irAEs compared to the standard chemotherapy groups.

**Conclusion:**

Nab-PTX/PTX+ICIs demonstrated an approach leading to decreased risk of irAEs compared with ICI monotherapy. This finding supports that ICIs+nab-PTX/PTX may be a safer treatment strategy. Moreover, we also found that the combination regimens containing ICIs had a higher risk of irAEs than standard chemotherapy. Additionally, ICIs+nab-PTX demonstrated a decreased risk of irAEs compared to ICIs+PTX. PD-1 inhibitors were associated with a higher risk of irAEs than PD-L1 inhibitors.

## 1 Introduction

Immune checkpoint inhibitors (ICIs) targeting programmed cell death 1 (PD-1), programmed cell death ligand 1 (PD-L1), or cytotoxic T lymphocyte antigen 4 (CTLA4), has been become one of the most important breakthroughs in cancer therapy (1). Immune suppression plays a key role in cancer growth and progression. ICIs promote immune responses against tumor cells by blocking immune checkpoint pathways. Treatment targeting immune checkpoints, such as anti-PD-1, anti-PD-L1, and anti-CTLA4 demonstrate impressive anti-tumor activities against several tumor types (2). Over the past decades, monoclonal antibodies against the PD-1/PD-L1 pathway have been approved for melanoma, prostate cancer, lung cancer, liver cancer, cervical cancer, gastric cancer, and breast cancer (3). However, a large proportion of patients do not respond or even resistant to ICIs (4–6). In several clinical trials (7–9) using biomarkers to predict the treatment response to anti–PD-1 or anti–PD-L1 therapies, the objective response rates were still unsatisfied (<50%).

Current research had been focused heavily on the improvement of the response rate of ICIs. Taxane-based chemotherapies, including albumin-bound paclitaxel (nab-PTX) and paclitaxel (PTX) (10–13), might have a “priming effect” for the immune system and improve the response to the ICIs (14). Although the “priming effect” of chemotherapy is still unexplained, ICIs combined with nab-PTX/PTX demonstrated superior efficacy in multiple clinical trials (13, 15). ICIs combined with nab-PTX/PTX has been widely adopted in the clinical practice (16), even though the combination therapy is not strongly recommended by the National Comprehensive Cancer Network (NCCN) (17–19).

Despite the substantial clinical benefits associated with ICIs+nab-PTX/PTX (20, 21), there has been rising concerns on the safety of combination therapy. ICIs may result in the activation of the immune system and are associated with adverse events, which are known as immune-related adverse events (irAEs) (22) (23, 24). The irAEs (22) include rash, colitis, hepatitis, hypothyroidism, hyperthyroidism and pneumonitis, occurring in up to 70% of patients treated with ICIs. The irAEs (22) could be severe or even fatal (22, 25), but their mechanism is still unclear. The NCCN has released several guidelines addressing adverse events associated with ICIs. For the combination of ICIs and nab-PTX/PTX, the synergistic effect of the combination strategy may attribute to therapy-associated cytokine release and T-cell-mediated organ infiltration (10, 11, 13, 26–30), leading to the alterations in safety profiles. However, the safety profiles of ICIs and nab-PTX/PTX is still inconsistent in the literature. Previous studies suggested that ICI alone is generally better tolerated than combination regimens (26, 31), but more recent studies concluded that ICIs and nab-PTX/PTX combination regimen demonstrated better safety profiles (32, 33). To our best knowledge, there are limited studies investigating the safety of ICIs+nab-PTX/PTX. Past meta-analyses mainly focused on a specific ICI or nab-PTX/PTX, failing to cover possible combination therapies (26, 34–36). With more ICIs on the market, it is very important to compare the safety profiles between combination regimens, but the head-to-head comparison is largely lacking. Therefore, we conducted a network meta-analysis (NMA) to comprehensively evaluate the safety profile and safety ranking of nab-PTX+ICI, PTX+ICI, ICI monotherapy and chemotherapy. This approach allowed us to combine direct and indirect evidence and rank the interventions based on their relative safety profiles.

## 2 Methods

This study was registered in the Prospective Register of Systematic Reviews (CRD42022326742). This NMA followed the Preferred Reporting Items for Systematic Reviews and Meta-Analysis (PRISMA) and the PRISMA extension statement for network meta-analysis (37).

### 2.1 Data sources and searches

We conducted a comprehensive search of relevant studies using keywords in electronic databases, including PubMed, Embase, Cochrane Library, Web of Science and ClinicalTrials.gov, between January 1, 2000 and August 15, 2022. Search key terms used in the search strategy include cancer, oncology, nab-PTX/PTX, immune checkpoint inhibitors, randomized controlled trials. The search strategy is described in the **Supplementary Table 1**. Two reviewers (WJ and JZ) firstly screened the titles and abstracts, then reviewed the full-texts of publications. Any discrepancies were resolved through discussion and consultation with the third reviewer (YW).

### 2.2 Study selection criteria

The study had pre-defined inclusion and exclusion criteria. Inclusion criteria were: (1) phase II or phase III randomized clinical trials (RCTs) with head-to-head comparison; (2) trials typically involve at least two arms the following mentioned: one ICI drug (PD-1/PD-L1 inhibitors), one ICI drug in combination with nab-PTX/PTX, one ICI drug in combination with chemotherapy; (3) study subjects diagnosed with cancer, (3) reported the incidence and grade of adverse events; (4) written in English.

The publications were excluded with any of the following: (1) letters, abstracts, reviews, posters, conference reports, unfinished studies or duplicated reports; (2) trials with insufficient data; (3) single-arm studies; (4) phase I randomized trials; (5) cost-effectiveness studies.

### 2.3 Data extraction

Two reviewers (WJ and JZ) extracted data independently, including first author, year of publication, treatment line, type of ICIs, stage of the cancer trial phase, treatment arm, incidence of grade 1-5 and grade 3-5 irAEs, sample size, patient age, sex distribution, cancer type, PD-L1 expression, Performance Status (PS) score, median follow-up time and Common Terminology Criteria for Adverse Events (CTCAE) edition.

In terms of adverse events data, because immune-related adverse events were the outcome of interest, we first evaluated “immune-related adverse events” in the main text and **Supplementary Materials** of published studies. We also screened all possible information available at ClinicalTrials.gov to obtain a more comprehensive data extraction. If irAEs were not available in the study (n=2), treatment-related adverse events were used and extracted.

### 2.4 Quality assessment

We used the Cochrane Collaboration’s risk of bias tool (38) to assess the quality of each trial included. Two reviewers (WH and YW) assessed on the 5 aspects, including the random sequence generation, allocation concealment, blinding, outcomes assessment, and reporting. Each aspect was graded based on the risk of bias, categorized by yes, no, or unclear. Any discrepancies in data extraction and quality assessment disagreements were resolved by discussion to achieve a consensus.

### 2.5 Statistical analysis

We summarized characteristics of trials, including first author, year of publication, treatment line, type of ICIs, stage of the cancer, trial phase, treatment arm, incidence of grade1-5 and grade 3-5 irAEs, sample size, patient age, sex distribution, cancer type, PD-L1 expression, PS score, median follow-up time and CTCAE edition. We accessed on the total number of all irAEs and the number of each specific irAEs, respectively. Incidence rates of grade 1-5 and grade 3-5 irAEs were compared among different treatment regimens, including chemotherapy, ICI monotherapy, ICI+nab-PTX and ICI+PTX. To investigate whether the occurrence of irAEs was influenced by the type of ICIs (PD-1 and PD-L1), we further subdivided the four treatment groups into six subgroups based on different types of ICIs: chemotherapy, PD-1 monotherapy, PD-L1 monotherapy, PD-1+PTX, PD-L1+PTX and ICIs+nab-PTX. Considering the relatively small sample sizes of PD-1+nab-PTX and PD-L1+nab-PTX, they were combined into one group.

To evaluate the statistical heterogeneity of the included trials, we accessed the I^2^ index and the Cochran Q statistic. Heterogeneity was defined as low for I^2^ values as 25–49%, moderate for 50–74%, and high for >75%, respectively. For NMA, we generated network plots depicting direct and indirect comparisons using STATA V.17.0. We used ADDIS-1.16.6 for head-to-head direct meta-analyses. To evaluate the risk of irAEs, we calculated ORs and 95% CIs using the random effects model, to account for unexplained heterogeneity. The random effects model is considered to be the most conservative method (39). Two-sided P<0.05 was considered significant.

Due to the potential low irAEs rate and limited sample size, irAEs may sometimes be rare (40, 41) or even absent. To address this issue, we used frequentists-framework-based network meta-analyses for all statistical analyses, and if there were no irAEs observed in a specific arm of a trial, the classic continuity correction of 0.5 for zero cells was applied for data preparation (26). Treatment effects were reported as the surface under the cumulative ranking curves (SUCRA). The higher SUCRA scores indicates a higher risk of irAEs.

Because consistency assessment is crucial in ensuring the robustness of direct and indirect comparison results (42), we used a two-step method to evaluate consistency. First, we used the loop‐specific approach to evaluate the presence of inconsistency from direct and indirect evidence (43). We calculated the inconsistency factors (IF) values, standard error of inconsistency factors (seIF) and p-value. If the 95% CI of IF contained ‘0’ and the p-value was higher than ‘0.05’, it was considered the direct evidence to be consistent with the indirect evidence. Second, we adopted node-splitting models to identify the consistency in the entire network on particular comparisons (nodes). P>0.05 indicated no significant inconsistency.

To evaluate the transitivity of the NMA, we compared the distribution of patient characteristics, aiming to ensure the similarity of the distribution of effect modifiers across different treatment comparisons in the network of trials. Furthermore, “comparison-adjusted” funnel plots were utilized to assess the presence small-sample effect and publication bias within the network of interventions.

## 3 Results

### 3.1 Study selection and patient characteristics

**Figure 1:**
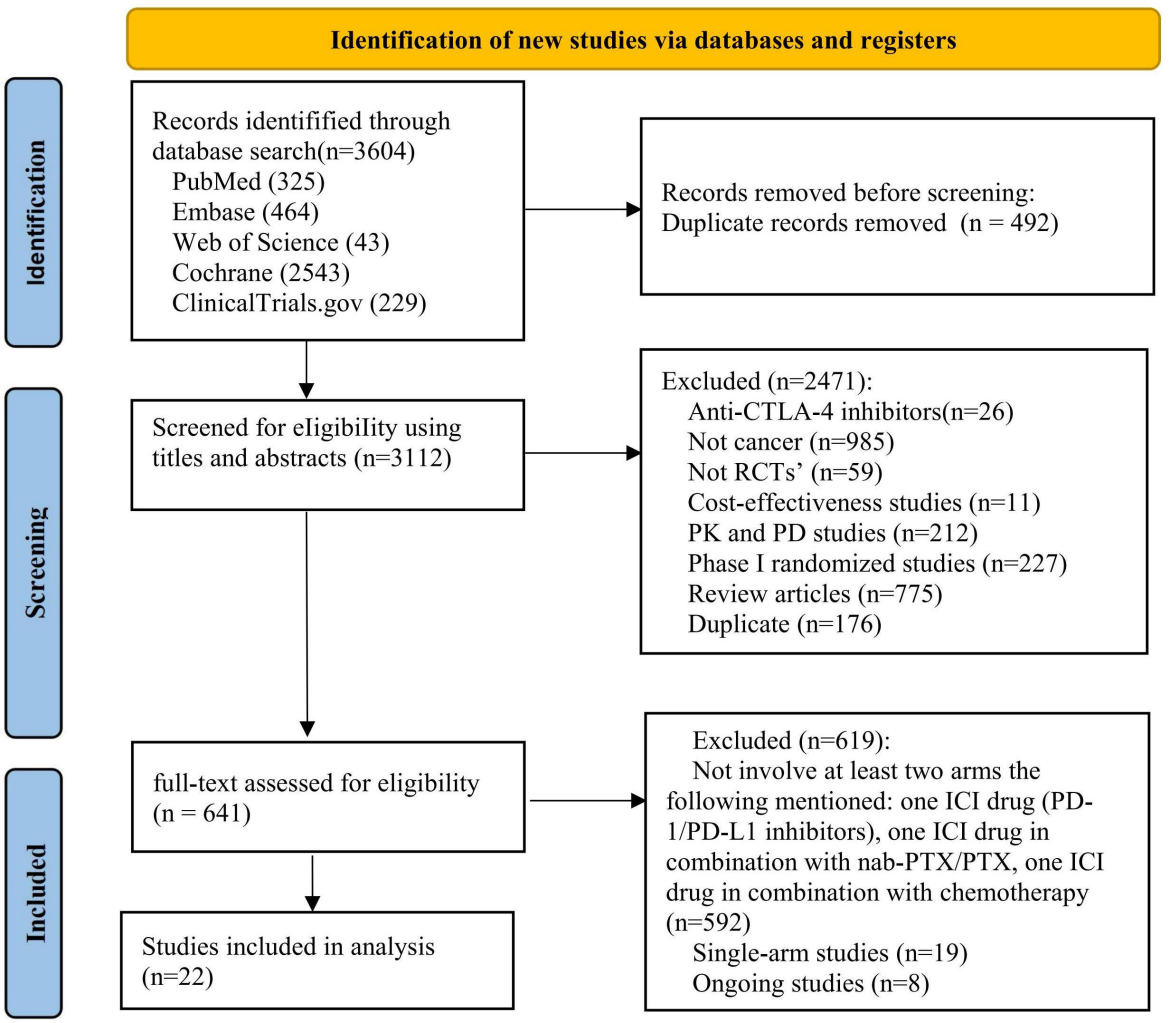
Flowchart of study selection followed PRISMA guidelines.

**Figure 2:**
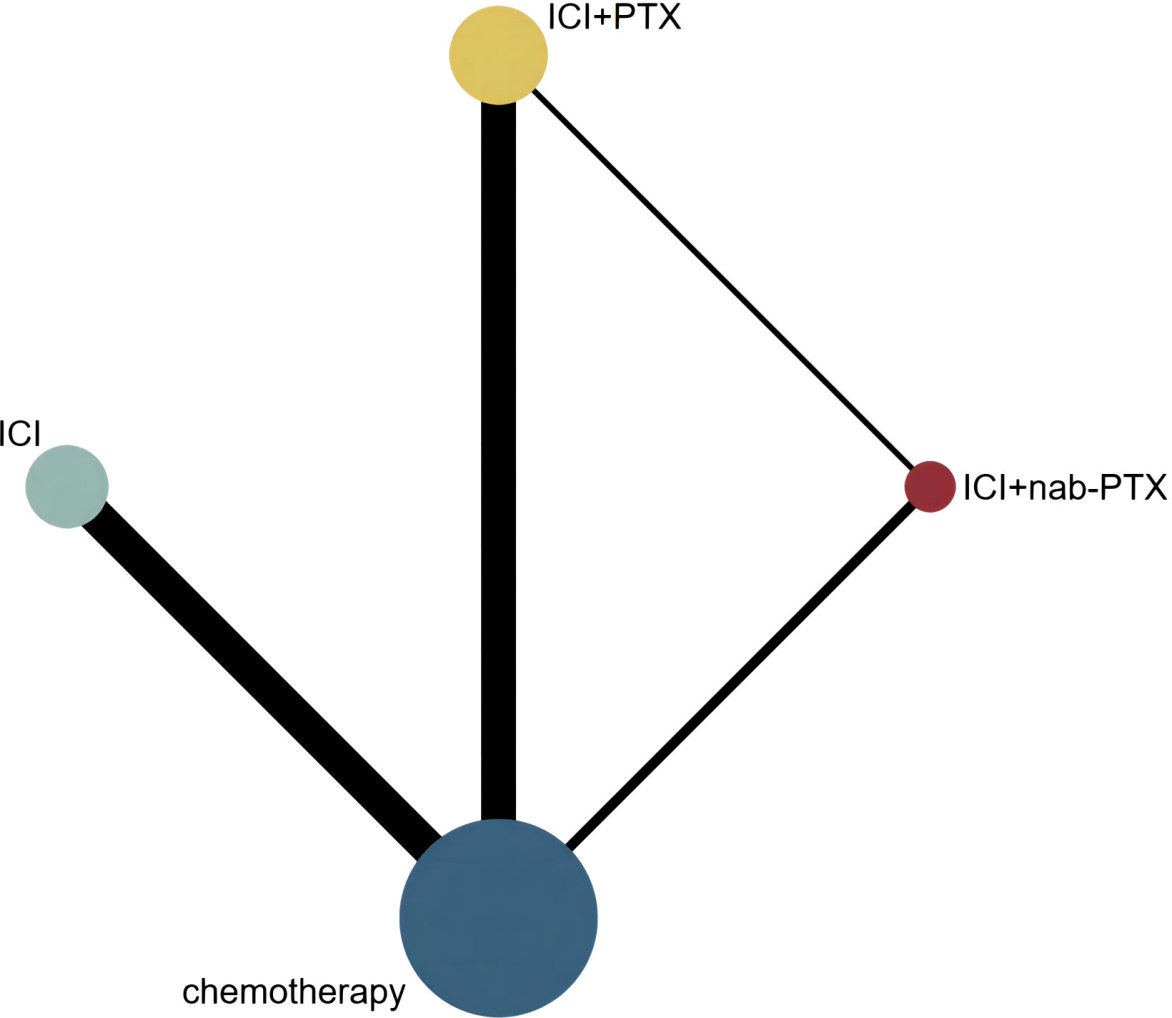
Network geometry of any event. The nodes in the figure represent the interventions being compared, while the edges represent the direct comparisons available between pairs of interventions (i.e. comparisons evaluated in at least one study). The node sizes are weighted based on the number of patients in each intervention arm, while the edges are weighted using inverse variance.

We identified 3 604 citations up to August 15, 2022, including 325 records from PubMed, 464 from Embase, 43 from Web of Science, 2 543 from Cochrane and 229 from ClinicalTrials.gov (**Figure 1**). After removing duplicates, 3 112 records were included in the titles and abstracts screening. A total of 641 publications underwent full-text review, after excluding 2 471 publications. Twenty-two RCTs (44–65) met the study selection criteria and were included in the analysis. **Figure 2** shows that among the patients included in the network meta-analysis, 3 919 patients received ICIs, 1 386 patients received ICI+nab-PTX, 3 302 patients received ICI+PTX, and 7 356 patients received chemotherapy.

**Table 1** show the information on the baseline characteristics of the included trials. There were 20 two arm trials, and only 2 studies have three arms. **Supplementary Table 2** displays the occurrence of irAEs in different treatment groups. all studies were phase III trials. Cancer types tested in these studies included lung cancer (n=12), breast cancer (n=3), urothelial cancer (n=3), ovarian cancer (n=2), gastric cancer (n=2). More detailed information can be found in **Supplementary Table 1**.

**Table 1:** Baseline characteristics of 22 studies.

First author, year	Patients	PD-1/ PD-L1	Treatment	Dosage	Stage	Line	Phase	Sample size	Median follow-up time (months)	Edition of CTCAE	PS 0-1	Famale	Age
Socinski MA, 2018	NSCLC	PD-L1	Ate+Bev+PTX + CBP	Ate (1200mg/3weeks)	IV	1	3	393	15.4	CTCAE 4.0	400	160	63
			Bev+PTX+CBP	PTX (200mg/m2/3weeks				394			400	161	63
Sugawara S, 2021	NSCLC	PD-1	Niv+Bev+PTX + CBP	Niv (360mg/200mg/m2)	IIIB/IV	1	3	273	13.7	CTCAE 4.0	275	70	64.1
			Placebo+Bev+PTX + CBP	PTX (200mg/m2/3weeks)				275			275	69	65.1
West H, 2019	NSCLC	PD-L1	Ate+Nab-PTX+CBP	Ate (1200mg/3weeks)	IV	1	3	473	18.5	CTCAE 4.0	482	206	66.1
			nab-PTX+CBP	nab-PTX (100mg/we)				232			239	102	67.1
Jotte R, 2020	NSCLC	PD-L1	Ate+CBP+nab-PTX	Ate (1200mg/3weeks)	IV	1	3	334	18.1	CTCAE 4.0	342	63	68.1
			Ate+CBP+PTX	PTX (200mg/m2/3weeks)				332			338	60	69.1
			CBP+nab-PTX	nab-PTX (100mg/we)				334			339	63	70.1
Moore KN, 2021	Ovarian Cancer	PD-L1	Ate+PTX+CBP+Bev	Ate (1200mg/3weeks)	III/IV	frontline	3	642	19.9	CTCAE 4.0		642	73.1
			Placebo+PTX+CBP+Bev	PTX (175mg/m2/3weeks)				644				644	74.1
Wang J, 2021	NSCLC	PD-1	Tis+CBP+PTX	Tis (200mg/3weeks)	IIIB/IV	1	3	120	8.6	CTCAE 5.0	120	13	75.1
			Tis+CBP+nab-PTX	nab-PTX (100mg/we)				119			119	7	76.1
			CBP+PTX	PTX (175mg/m2/3weeks)				121			121	10	77.1
Monk BJ, 2021	Epithelial Ovarian Cancer	PD-1	Ave+PTX+CBP	Ave (10mg/kg/2weeks)	III/IV	frontline	3	329	29	CTCAE 4.0	329	331	80.1
			PTX+CBP	PTX (175mg/m2/3weeks)				334			332	335	81.1
Hellmann MD, 2018	NSCLC	PD-1	Nivoluma	Niv (240mg/2weeks)	IV	1	3	391	min 11.2	CTCAE 4.0	394	124	64
			Platinum doublet chemotherapy					570			577	198	64
Herbst RS, 2020	NSCLC	PD-L1	Ate	Ate (1200mg/3weeks)	IV	1	3	286	54.5	CTCAE 4.0	285	87	64
			platinum-based chemotherapy					263			287	89	65
Powles T, 2021	Urothelia Cancer	PD-1	Pem	Pem (200mg/3weeks)	locally advanced, unresectable, or metastati	1	3	302	31.7	CTCAE 4.0	282	79	67
			Standard-of-care Chemotherapy					342			330	90	68
Powles T, 2020	Urothelia Cancer	PD-L1	Dur	Dur (1500mg/4weeks)	unresectable, locally advanced or metastati	1	3	345	41.2	CTCAE 4.0	346	97	67
			Standard of Care Chemotherapy					313			344	70	68
Sezer A, 2021	NSCLC	PD-1	Cem	Cem (350mg/3weeks)	IIIB/IIIC/IV	1	3	355	32	CTCAE 4.0	356	44	63
			Standard-of-care Chemotherapy					342			354	60	64
Rizvi NA, 2020	NSCLC	PD-L1	Dur	Dur (20mg/kg/4weeks)	IV	1	3	369	40	CTCAE 4.0	372	118	63.2
			Standard-of-care Chemotherapy					352			370	122	63.6
Shitara K, 2020	Gastric Cancer	PD-1	Pem	Pem (200mg/3weeks)	locally advanced/unresectable or metastati	1	3	254	29.4	CTCAE 4.0	265	76	61
			Placebo +Standard-of-care Chemotherapy					244			250	71	62.5
Reck M, 2021	NSCLC	PD-1	Pem	Pem (200mg/3weeks)	IV	1	3	154	60		153	62	64.5
			platinum-based chemotherapy					151			151	56	66
Mok TSK, 2019	NSCLC	PD-1	Pem	Pem (200mg/3weeks)	locally advanced or metastati	1	3	636	12.8	CTCAE 4.0	637	187	63
			platinum-based chemotherapy					615			637	185	63
Carbone DP, 2017	NSCLC	PD-L1	Niv	Niv (3mg/kg/2weeks)	IV	1	3	267	13.5	CTCAE 4.0	268	87	63
			platinum-based chemotherapy					263			269	122	65
Emens LA, 2021	Triple-Negative Breast Cancer	PD-L1	Ate plus nab-PTX	Ate (840mg/2 weeks)	locally advanced, or metastati	1	3	460	18.8	CTCAE 4.0	450	448	55
			nab-PTX+ placebo	nab-PTX (100mg/we)				430			450	450	56
Miles D, 2021	Triple-Negative Breast Cancer	PD-L1	Ate+PTX	Ate (840mg/2weeks)	metastatic or unresectable locally advance	1	3	432	14.2	CTCAE 4.0	431	430	54
			placebo+PTX	PTX (90 mg/m2/we)				217	14.5		220	220	53
Schmid P, 2020	Triple-Negative Breast Cancer	PD-1	Pem+ PTX+ CBP	Pem (200mg/3weeks)	II/III	1	3	781	15.5	CTCAE 4.0	784	783	49
			Placebo+ PTX+CBP	PTX (80 mg/m2/we)				389			390	390	48
Shitara K, 2018	gastric or gastro-oesophageal junction cancer	PD-1	Pem	Pem (200mg/3weeks)	advance	2	3	294	7.9	CTCAE 4.0	296	94	62.5
			PTX	PTX (standard-dos)				276			295	88	60
Bellmunt J, 2017	Urothelial Cancer	PD-1	Pem	Pem (200mg/3weeks)	advance	2	3	266	14.1	CTCAE 4.0	262	70	67
			Chemotherapy					255			264	70	65

NSCLC, non-small cell lung cancer; PTX, Paclitaxel; nab-PTX, nanoparticle albumin-bound paclitaxel; Ate, Atezolizumab; Tis, Tislelizumab; AVE, Avelumab; Niv, Nivolumab; Pem, Pembrolizumab; Dur, Durvalumab; Cem, Cemiplimab; Bev,Bevacizumab; CBP, Carboplatin.

**Figure 3:**
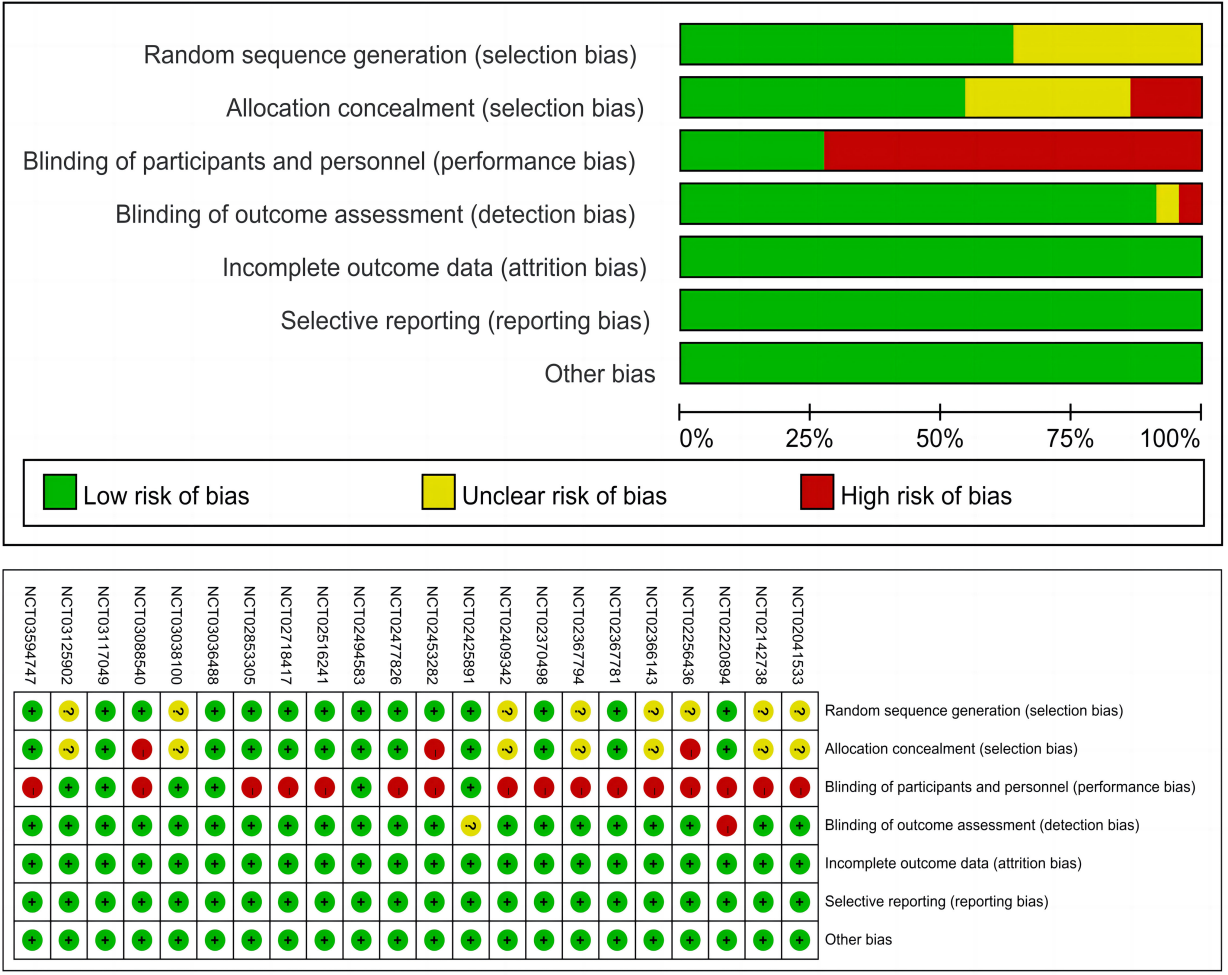
Risk of bias assessment for the 22 included randomized controlled trials.

**Figure 3** presents the risk of bias summary for the included trials. It is worth noting that many trials were open-labeled due to the differences in infusion duration, administration schedules, and premedication requirements for immune checkpoint inhibitors, which would make masking difficult, but this does not indicate that the studies were of low quality.

### 3.2 Heterogeneity, inconsistency, and transitivity assessment

**Figure 4:**
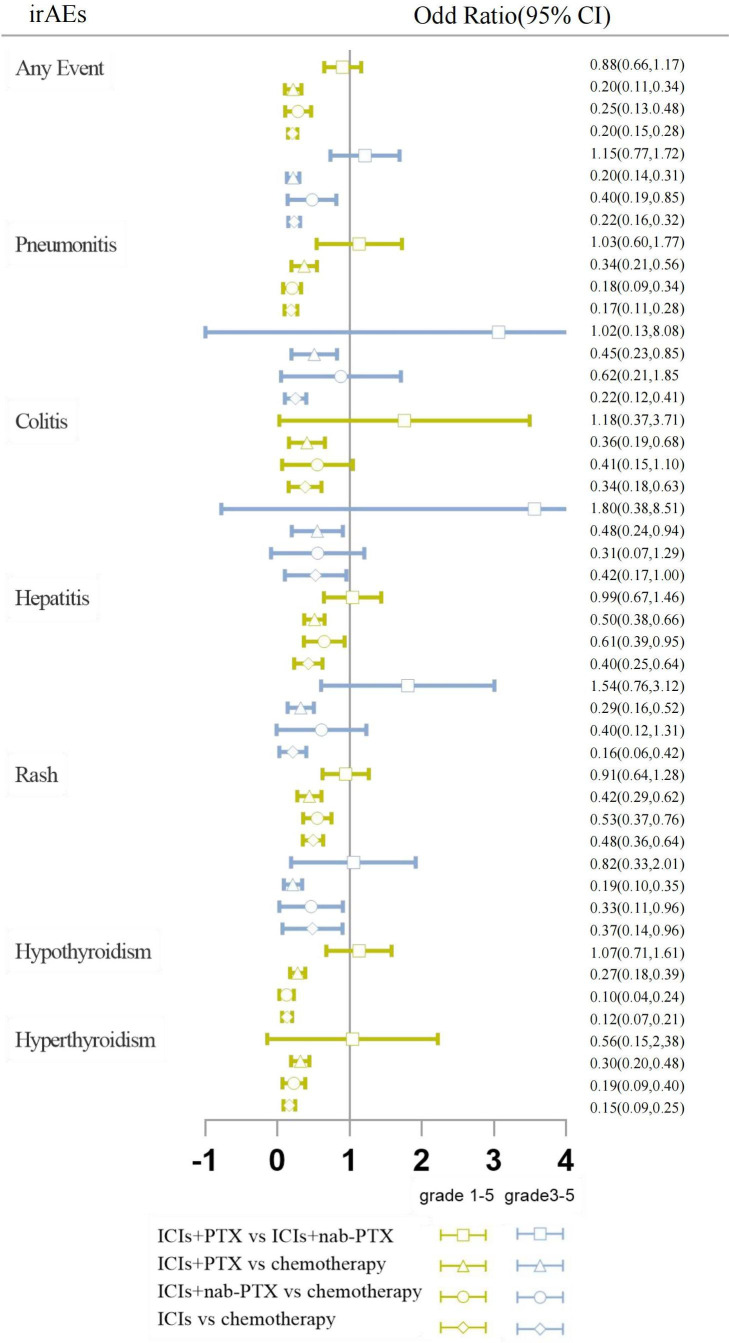
Forest plots results of head-to-head comparisons. The results are presented as odds ratios (ORs) with corresponding 95% confidence intervals (CIs). The vertical line represents the null effect, which is set at 1. The horizontal line depicts the CIs, and the hollow shape represents the point estimate, summarizing the ORs. When interpreting the forest plot for each pairwise comparison, it should be noted that if the hollow shape with the CI does not intersect with the vertical line of null effect, a statistically significant difference is observed. If the CI is on the left of the null effect, the event is significantly higher in the intervention arm, while if the CI is on the right, the event is statistically more frequent in the reference arm. If the CI intersects with the line of null effect, the difference between the two procedures is not statistically significant.

Pairwise comparisons with heterogeneity estimates are presented in **Figure 4** and **Supplementary Figure 1**. Nearly all comparisons indicating low heterogeneity. Inconsistency analysis using node-splitting and loop-specific approaches showed no significant inconsistency between direct and indirect analyses. Results of the inconsistency evaluation are presented in **Supplementary Tables 6, 7**. All included clinical trials enrolled cancer patients; all the trials were phase III RCTs; utilizing standard doses (the dosage of PTX in the Asian population was 175 mg/m^2^/3 weeks, and of other races was 200mg/m^2^/3 weeks); median follow-up time was 23.2 months (ranging from 7.9 to 60 months); patients were at advanced stages of cancer, PS scores were mostly 0-1, and age characteristics were similar. By comparison, the baseline characteristic distribution of each treatment group was balanced, indicating acceptable transitivity. The network’s funnel plots visually indicate potential publication bias, and no significant asymmetry was observed (**Supplementary Figure 6**)

### 3.3 Comparison of irAEs

The network geometry and the contribution plots are reported in **Supplement Figures 2, 3**. While head-to-head direct meta-analyses are shown in **Figure 4** and **Supplementary Figure 1**.

Based on the consistency model, the ORs for pairwise comparisons of irAEs are shown in **Table 2**. Almost all treatment regimens showed statistically significant differences with the chemotherapy group. The ICI+PTX had a significantly lower risk of grade 1-5 hyperthyroidism (OR=0.46, 95% CrI: 0.22-0.96) and grade 1-5 hypothyroidism (OR=0.49, 95% CrI: 0.26-0.93) than ICIs. Notably, comparing with ICI monotherapy, ICI+nab-PTX was associated with a decreased risk of grade 3-5 pneumonitis (OR=0.28, 95% CrI: 0.09,0.90).

**Table 2:** The odds ratios (ORs) for pairwise comparisons of irAEs based on network consistency model.

Grade 3-5 Pneumonitis				Grade 3-5 Colitis			
ICI+nab-PTX	1.46 (0.59,3.60)	**3.61 (1.12,11.67)**	0.68 (0.26,1.74)	ICI+nab-PTX	0.57 (0.17,1.87)	0.58 (0.13,2.65)	**0.26 (0.08,0.87)**
1.50 (0.70,3.20)	ICI+PTX	2.48 (0.99,6.21)	**0.46 (0.25,0.85)**	0.89 (0.61,1.31)	ICI+PTX	1.02 (0.33,3.18)	**0.47 (0.25,0.88)**
0.73 (0.28,1.87)	0.49 (0.22,1.07)	ICI	**0.19 (0.09,0.37)**	0.65 (0.33,1.29)	0.73 (0.39,1.37)	ICI	0.46 (0.18,1.16)
**5.05 (2.37,10.78)**	**3.37 (1.97,5.77)**	**6.93 (3.89,12.35)**	chemotherapy	**1.71 (1.21,2.42)**	**1.92 (1.42,2.60)**	**2.64 (1.51,4.61)**	chemotherapy
Grade 1-5 Pneumonitis				Grade 1-5 Colitis			
Grade 3-5 Hepatitis				Grade 3-5 Rash			
ICI+nab-PTX	1.11 (0.43,2.86)	2.17 (0.55,8.56)	**0.34 (0.14,0.79)**	ICI+nab-PTX	1.43 (0.64,3.20)	0.76 (0.20,2.92)	**0.24 (0.10,0.57)**
0.89 (0.61,1.31)	ICI+PTX	1.96 (0.53,7.32)	**0.30 (0.14,0.64)**	0.85 (0.55,1.31)	ICI+PTX	0.53 (0.17,1.69)	**0.17 (0.10,0.28)**
0.65 (0.33,1.29)	0.73 (0.39,1.37)	ICI	**0.15 (0.05,0.45)**	0.89 (0.51,1.57)	1.05 (0.65,1.71)	ICI	**0.32 (0.11,0.89)**
**1.71 (1.21,2.42)**	**1.92 (1.42,2.60)**	**2.64 (1.51,4.61)**	chemotherapy	**1.88 (1.25,2.85)**	**2.23 (1.68,2.96)**	**2.11 (1.43,3.12)**	chemotherapy
Grade 1-5 Hepatitis				Grade 1-5 Rash			
Grade 1-5 Hyperthyroidism				Grade 1-5 Any Event			
ICI+nab-PTX	0.95 (0.50,1.80)	2.08 (0.89,4.90)	**0.26 (0.13,0.50)**	ICI+nab-PTX	1.23 (0.62,2.44)	1.33 (0.63,2.85)	**0.26 (0.14,0.48)**
1.48 (0.80,2.75)	ICI+PTX	**2.20 (1.04,4.64)**	**0.27 (0.16,0.46)**	0.65 (0.36,1.18)	ICI+PTX	1.08 (0.56,2.08)	**0.21 (0.13,0.34)**
0.72 (0.33,1.57)	**0.49 (0.26,0.93)**	ICI	**0.13 (0.07,0.22)**	0.59 (0.28,1.24)	0.91 (0.47,1.77)	ICI	**0.19 (0.12,0.31)**
**6.04 (3.08,11.85)**	**4.09 (2.61,6.40)**	**8.39 (4.98,14.15)**	chemotherapy	**2.90 (1.67,5.03)**	**4.48 (2.90,6.92)**	**4.93 (2.96,8.20)**	chemotherapy
Grade 3-5 Hyperthyroidism				Grade 3-5 Any Event			

This is an indirect comparison of adverse events of grades 1-5 and 3-5 in different treatment regimens. The combined odds ratios and 95% confidence intervals indicate the results between the highest and lowest treatment regimens. Each unit contains the combined odds ratio and 95% confidence interval, with significant results highlighted in thick lines.

The ranking analysis performed with SUCRA provided a ranking of each treatment group based on the incidence of irAEs, as shown in **Table 3**. The ICIs was associated with the worst safety ranking for grade 1-5 of any event (probability=79.3%), followed by ICI+PTX (70.8%), ICI+nab-PTX (49.9%), and finally chemotherapy (0%). The safety ranking for grade 3-5 of any adverse event was the same to irAEs: ICIs (84.6%), ICI+PTX (76.8%), ICI+nab-PTX (38.6%), and chemotherapy (0%). In addition, compared to the other three treatment groups, ICI monotherapy had the highest risk for causing pneumonitis (grade 1-5 and grade 3-5), colitis (grade 1-5), hepatitis (grade 1-5 and grade 3-5), hypothyroidism (grade 1-5) and hyperthyroidism (grade 3-5). The main irAEs caused by ICI+PTX was rare. ICI+nab-PTX mainly caused grade 3-5 colitis. Additionally, ICIs+nab-PTX has a lower risk of irAEs than ICIs+PTX. More detailed information can be found in **Supplementary Table 4** and **Supplementary Figures 4, 5**.

**Table 3:** Pooled results of toxicity spectra and SUCRA rankings based on each specific irAEs.

(A)	1	2	3	4		
Grade 1-5 Any Event	ICI (79.3)	ICI+PTX (70.8)	ICI+nab-PTX (49.9)	Chemotherapy (0.0)		
Grade 3-5 Any Event	ICI (84.6)	ICI+PTX (76.8)	ICI+nab-PTX (38.6)	Chemotherapy (0.0)		
Grade 1-5 Pneumonitis	ICI (90.4)	ICI+nab-PTX (70.2)	ICI+PTX (39.4)	Chemotherapy (0.0)		
Grade 3-5 Pneumonitis	ICI (98.6)	ICI+PTX (60.6)	ICI+nab-PTX (33.6)	Chemotherapy (7.3)		
Grade 1-5 Colitis	ICI (78.1)	ICI+nab-PTX (64.6)	ICI+PTX (57.0)	Chemotherapy (0.3)		
Grade 3-5 Colitis	ICI+nab-PTX (85.4)	ICI (57.3)	ICI+PTX (54.8)	Chemotherapy (2.5)		
Grade 1-5 Hepatitis	ICI (91.2)	ICI+PTX (62.9)	ICI+nab-PTX (45.8)	Chemotherapy (0.1)		
Grade 3-5 Hepatitis	ICI (90.4)	ICI+PTX (57.9)	ICI+nab-PTX (51.4)	Chemotherapy (0.2)		
Grade 1-5 Rash	ICI+PTX (78.3)	ICI (69.5)	ICI+nab-PTX (52.2)	Chemotherapy (0.0)		
Grade 3-5 Rash	ICI+PTX (88.9)	ICI+nab-PTX (61.5)	ICI (49.2)	Chemotherapy (0.5)		
Grade 1-5 Hypothyroidism	ICI (92.8)	ICI+nab-PTX (69.8)	ICI+PTX (37.5)	Chemotherapy (0.0)		
Grade 1-5 Hyperthyroidism	ICI (97.8)	ICI+nab-PTX (53.6)	ICI+PTX (48.6)	Chemotherapy (0.0)		
(B)	1	2	3	4	5	6
Grade 1-5 Any Event	PD-1+PTX (87.0)	PD-1 (69.7)	PD-L1 (58.6)	ICI+nab-PTX (45.9)	PD-L1+PTX (39.5)	Chemotherapy (0.0)
Grade 3-5 Any Event	PD-1+PTX (87.0)	PD-L1 (73.0)	PD-1 (65.7)	PD-L1+PTX (43.1)	ICI+nab-PTX (31.2)	Chemotherapy (0.0)
Grade 1-5 Pneumonitis	PD-1 (96.4)	ICI+nab-PTX (68.5)	PD-L1 (48.7)	PD-L1+PTX (47.4)	PD-1+PTX (38.8)	Chemotherapy (0.3)
Grade 3-5 Pneumonitis	PD-1 (90.6)	PD-L1+PTX (65.7)	PD-L1 (64.8)	PD-1+PTX (40.7)	ICI+nab-PTX (34.1)	Chemotherapy (4.0)
Grade 1-5 Colitis	PD-L1 (79.8)	PD-1+PTX (77.9)	PD-1 (57.7)	ICI+nab-PTX (48.5)	PD-L1+PTX (35.2)	Chemotherapy (1.0)
Grade 3-5 Colitis	PD-1+PTX (87.6)	ICI+nab-PTX (65.2)	PD-L1 (54.2)	PD-1 (54.0)	PD-L1+PTX (31.5)	Chemotherapy (7.5)
Grade 1-5 Hepatitis	PD-1 (90.2)	PD-1+PTX (79.0)	PD-L1 (52.7)	PD-L1+PTX (45.8)	ICI+nab-PTX (31.7)	Chemotherapy (0.6)
Grade 3-5 Hepatitis	PD-1+PTX (74.4)	PD-1 (73.3)	PD-L1 (70.3)	ICI+nab-PTX (41.9)	PD-L1+PTX (39.0)	Chemotherapy (1.2)
Grade 1-5 Rash	PD-1+PTX (94.9)	PD-1 (67.0)	PD-L1 (49.1)	PD-L1+PTX (46.2)	ICI+nab-PTX (42.7)	Chemotherapy (0.2)
Grade 3-5 Rash	PD-1+PTX (88.5)	PD-1 (70.2)	PD-L1+PTX (57.2)	ICI+nab-PTX (51.0)	PD-L1 (31.0)	Chemotherapy (2.2)
Grade 1-5 Hypothyroidism	PD-L1 (87.8)	PD-1 (75.0)	ICI+nab-PTX (62.1)	PD-1+PTX (47.9)	PD-L1+PTX (27.2)	Chemotherapy (0.0)
Grade 1-5 Hyperthyroidism	PD-1 (89.6)	PD-L1 (71.8)	PD-1+PTX (67.3)	ICI+nab-PTX (42.7)	PD-L1+PTX (28.5)	Chemotherapy (0.0)

The number in each bracket indicates the probability of risk ranking. (A) If ICI+nab-PTX has a higher ranking than ICI+PTX, the squares are shown with a yellow background. Otherwise, they are displayed on a blue background. (B) If the treatment groups containing PD-1 have a higher ranking than those with PD-L1, the squares are shown with a yellow background. Otherwise, they are displayed on a blue background.

### 3.4 Comparison of irAEs between PD-1 and PD-L1

The network geometry and the contribution plots are reported in **Supplement Figures 2, 3**. While head-to-head direct meta-analyses are shown in **Supplementary Figure 1**. **Supplementary Table 3** presents the safety profiles of six treatment groups. For grade 1-5 irAEs, all treatment groups were associated with statistically significantly higher risks compared with chemotherapy. PD-1+PTX was associated with a statistically significantly lower risk of pneumonitis compared to PD-1 (OR=0.32, 95% CrI: 0.11-0.92). PD-L1+PTX showed a noticeably lower risk of rash compared to PD-1+PTX (OR=0.52, 95% CrI: 0.28-0.98). Additionally, PD-L1+PTX also presented a lower risk of hypothyroidism compared to PD-L1(OR=0.34, 95% CrI: 0.12-1.00). Of note, adding PTX to the treatment regimens can increase the risk of pneumonitis and hypothyroidism. Secondly, PD-1 showed a significantly higher risk than PD-L1.

Statistically substantial differences were observed only when comparing with chemotherapy groups for grade 3-5 irAEs. The ranking probability is presents in **Table 3B**, **Supplementary Table 5** and **Supplementary Figure 4, 5**. The treatment groups containing PD-1 exhibited a higher risk of adverse reactions than those containing PD-L1.

## 4 Discussion

Immunotherapy has revolutionized treatment approaches for cancer, with ICIs combined with chemotherapy have shown remarkable clinical benefits, particularly the wide used nab-PTX/PTX combination strategies (13, 66). This large NMA is based on 22 RCTs including 15 963 patients. To our knowledge, this study is the first NMA that includes all cancers and provide important safety ranking of four treatment regimens involving ICI+ nab-PTX/PTX and a comparison of the safety profiles between PD-1 and PD-L1 inhibitors are provided as valuable references for clinical practice.

The nab-PTX was associated with unique advantages, such as without the use of a solvent, faster and greater tissue penetration, and slower elimination compared to PTX, which has made nab-PTX as the preferred option for combination therapy in the clinical settings (13). However, some studies have indicated that immunetherapy+chemotherapy may decrease the risk of irAEs (32, 33), while others have reached the opposite conclusion (32, 33, 67, 68). In our analysis, we found that the specific combination regimen of nab-PTX/PTX+ICIs is a safer therapeutic approach, significantly reducing the risk of irAEs occurrence. Moreover, nab-PTX demonstrates superior safety compared to PTX

The immune-related pneumonitis was associated with treatment discontinuation and mortality (69, 70). In this analysis, we specifically evaluated the immune-related pneumonitis. We found ICI monotherapy was linked to a higher risk of grade 3-5 immune‐related pneumonitis compared to nab-PTX+ICI, and comparing with PD-1, PD-1+PTX was associated with a statistically significant lower risk of grade 1-5 pneumonitis. In addition, ICI therapy was found to be associated with increased risks of grade 1-5 hypothyroidism and hyperthyroidism compared with PTX+ICI, while PD-L1+PTX presented a lower risk of hypothyroidism compared to PD-L1. In addition, according to the ranking results of irAEs, nab-PTX/PTX+ICI has a better safety profile than ICIs monotherapy for most irAEs, potentially reducing the risk of irAEs associated with ICIs. One possible reason is that in phase III clinical trials, the use of cytotoxic chemotherapy drugs is close to the maximum tolerated dose, leading to immune-suppressive (71). Consequently, the incidence of adverse events is low. Additionally, patients receiving PTX need to be pretreated with corticosteroids to prevent hypersensitivity (72), which can also suppress the immune system, reduce inflammation, and alleviate the development of irAEs (73). There may be other underlying mechanisms contributing to this phenomenon that require further investigation in basic research. In summary, our findings suggest that combining nab-PTX/PTX with ICIs offers a safer clinical treatment strategy.

For immune-related rash, PD-1+PTX was found to significantly increase the risk of grade 1-5 rash compared with PD-L1+PTX. In addition, according to the ranking of adverse reactions, our analysis found that the group containing PD-1 had s higher risk of irAEs compared to the group containing PD-L1, which is consistent with the previous research results (32, 74–76). In contrast, PD-1 antibody can simultaneously block the binding of PD-1 to both PD-L1 and PD-L2, resulting in a more comprehensive inhibition of the immune escape pathway and a higher incidence of irAEs (77). A previous study has reported that the competitive binding of PD-1 antibody to PD-L2 can disrupt the normal function of PD-L2 and other binding partners, leading to the activation of RGMb (repulsive guidance molecule) and subsequently cause pneumonitis (78). A recent study has demonstrated that exosomes derived from melanoma cells also express PD-L1. These exosomes, which contain PD-L1, travel through the bloodstream and can directly bind to the PD-1 receptor on the surface of T cells, resulting in T cell dysfunction. As a result, PD-L1 antibodies may be rendered ineffective by exosomal-PD-L1 before reaching the tumor cells. However, this issue does not arise with PD-1 antibodies, as they bind to the PD-1 receptor on T cells and exosomal-PD-L1 cannot neutralize their effects (79). These reasons may all contribute to the enhanced safety of PD-L1 inhibitors compared to PD-1 inhibitors. Numerous basic studies have reported a synergistic effect of taxane-combined immunotherapy (29, 80–82). This study further supports the potential benefits of this strategy in reducing the incidence of irAEs, providing a valuable guidance for clinical decision-making and serving as an evidence-based foundation for further basic research. However, there is still a lack of evidence of direct comparison. Therefore, further prospective RCTs and detailed basic research are needed to enrich the evidence.

Our study has several limitations. Frist, a standardized diagnostic criteria for irAEs is still lacking. In this study, irAEs data were extracted from “immune-related adverse events”. To obtain a more robust estimate of safety profile, we also extracted “treatment-related adverse events. In addition, although CTCAE 4.0 was the main version of adverse event evaluation criteria in all trials, we could not exclude the possibility that the different judgment criteria and grading strategies have been applied in the evaluation of irAEs. Second, the median follow-up time was varied among the trials included in the analysis, and it is possible that the reporting of irAEs with late-onset might be varied greatly. Third, the expression level of PD-L1 has been recognized as a potentially important and clinically valuable indicator for anti-PD-1 or anti-PD-L1 treatment (83, 84), but most trials failed to provide this important information. Fourth, the limited sample size of arms containing nab-PTX also prevents subgroup analysis, and the results involving nab-PTX should be interpreted with caution. Finally, for zero-events in any arm, STATA replaced them with the default value of 0.5, which increased the sample size per treatment by 1.

## 5 Conclusion

Our findings demonstrate that this combination therapies can significantly reduce the risk of immune-related adverse events, providing robust evidence to address the current controversial academic issues and offering clinical decision-making guidance for cancer patients. Furthermore, this study also confirms previous research findings that anti-PD-L1 inhibitors are safer than anti-PD-1 inhibitors and it demonstrates ICIs+nab-PTX has a lower risk of irAEs occurrence than ICIs+PTX.

## Author contributions

Conception and design: WH, JY, and WC. Wrote the manuscript: WH. Acquired data: WH, JZ and YW. Analyzed the data: WH. Discussed the results and implications of findings: WH, JZ, YW, BF, and SJ. Drafting of the manuscript: WH. Review and editing: JY and WC. All authors contributed to the article and approved the submitted version.
