# Correction: Immune-related adverse events associated with nab-paclitaxel/paclitaxel combined with immune checkpoint inhibitors: a systematic review and network meta-analysis

**DOI:** 10.3389/fimmu.2026.1812769

**Published:** 2026-03-05

**Authors:** Wenjing Hao, Jun Zhang, Yunxia Wang, Boyu Fang, Shasha Jin, Jing Yuan, Weimin Cai

**Affiliations:** 1 Department of Clinical Pharmacy and Pharmacy Administration, School of Pharmacy, Fudan University, Shanghai, China; 2 School of Pharmacy, Minhang Hospital, Fudan University, Shanghai, China

**Keywords:** immune-related adverse events, systematic literature review, network meta-analysis, immune checkpoint inhibitors, nab-paclitaxel, paclitaxel

In the published article, there was an error in the **Funding** statement. “This work was supported by grant from the National Natural Science Foundation of China [Grant No. 8217130423]”.

The correct **Funding** statement appears below.

“This work was supported by grant from the National Natural Science Foundation of China [Grant No. 82173892]“

The authors apologize for this error and state that this does not change the scientific conclusions of the article in any way.

The original article has been updated.

