# Supplementary material for: Immune-related adverse events associated with nab-paclitaxel/paclitaxel combined with immune checkpoint inhibitors: a systematic review and network meta-analysis
Source: Front Immunol. 2023 Jul 14;14:1175809. doi: 10.3389/fimmu.2023.1175809 (PMC10375236; doi:10.3389/fimmu.2023.1175809)
Supplement: Supplementary file 1 [file DataSheet_1.pdf]

Supplementary Material

Immune-related adverse events associated with nab-paclitaxel/paclitaxel combined with immune checkpoint inhibitors: a systematic review and network meta-analysis

Wenjing Hao, Jun Zhang, Yunxia Wang, Boyu Fang, Jing Yuan* and Weimin Cai*

Department of Clinical Pharmacy, School of Pharmacy, Fudan University, Shanghai, China,

*** Correspondence:**

Weimin Cai: weimincai@fudan.edu.cn

Jing Yuan: jyuan@fudan.edu.cn

1 Supplementary Tables and Figures

1.1 Supplementary Tables

First author, year	Treatment	Smoking status			PD-L1 expression level			PD-L1 evaluation method
		Never	Current	Previous	>50%	1%-49%	<1%	
Socinski MA, 2018	Ate+Bev+PTX + CBP	82	90	228	166	217	16	VENTANA SP142
	Bev+PTX+CBP	77	92	231	148	231	21	
Sugawara S, 2021	Niv+Bev+PTX + CBP	61	18	196	73	82	120	IHC 28-8 pharmDx kit
	Placebo+Bev+PTX + CBP	54	21	200	74	81	120	
West H, 2019	Ate+Nab-PTX+CBP	64	96	323	91	139	253	VENTANA SP142
	nab-PTX+CBP	20	53	167	43	68	129	
Jotte R, 2020	Ate+CBP+nab-PTX	32	311		47	136	160	VENTANA SP142
	Ate+CBP+PTX	30	308		48	120	170	
	CBP+nab-PTX	23	316		44	125	171	
Moore KN, 2021	Ate+PTX+CBP+Bev				391		260	VENTANA SP142
	Placebo+PTX+CBP+Bev				393		257	
Wang J, 2021	Tis+CBP+PTX	24	96		42	30	48	VENTANA SP263
	Tis+CBP+nab-PTX	12	107		42	30	47	
	CBP+PTX	23	98		41	31	49	
Monk BJ, 2021	Ave+PTX+CBP				160		171	VENTANA SP263
	PTX+CBP				169		166	
Hellmann MD, 2018	Nivolumab	7	130		101		38	IHC 28-8 pharmDx kit
	Platinum doublet chemotherapy	11	149		112		48	
Herbst RS, 2020	Ate	34	74	166	107	170	0	VENTANA SP142
	platinum-based chemotherapy	35	81	161	98	179	0	
Powles T, 2021	Pem							IHC 22C3 pharmDx assay
	Standard-of-care Chemotherapy							
Powles T, 2020	Dur	125	60	159	209	137	0	VENTANA SP263
	Standard of Care Chemotherapy	101	61	178	207	137	0	
Sezer A, 2021	Cem		133	223	283			IHC 22C3 pharmDx assay
	Standard-of-care Chemotherapy		120	234	280			
Rizvi NA, 2020	Dur	24	92	47	118	61	95	VENTANA SP263
	Standard-of-care Chemotherapy	21	102	39	107	82	83	
Shitara K, 2020	Pem							IHC 22C3 pharmDx assay
	Placebo +Standard-of-care Chemotherapy							
Reck M, 2021	Pem	5	34	115	154	0	0	IHC
	platinum-based chemotherapy	19	31	101	151	0	0	
Mok TSK, 2019	Pem	142	125	370	299	338	0	IHC 22C3 pharmDx assay
	platinum-based chemotherapy	140	146	351	337	300	0	
Carbone DP, 2017	Niv	30	52	186	88	208	0	IHC 28-8 pharmDx kit
	platinum-based chemotherapy	29	55	182	126	210	0	
Emens LA, 2021	Ate plus nab-PTX				185		266	VENTANA SP142
	nab-PTX+ placebo				184		267	
Miles D, 2021	Ate+PTX				191		240	VENTANA SP142
	placebo+PTX				101		220	
Schmid P, 2020	Pem+ PTX+ CBP				656		127	IHC 22C3 pharmDx assay
	Placebo+ PTX+CBP				317		69	
Shitara K, 2018	Pem				196		99	IHC 22C3 pharmDx assay
	PTX				199		96	
Bellmunt J, 2017	Pem				74	186		IHC 22C3 pharmDx assay
	Chemotherapy				90	176		

Supplementary Table 1. Baseline characteristics of 22 trials

NSCLC, non-small cell lung cancer; PTX, Paclitaxel; nab-PTX, nanoparticle albumin-bound paclitaxel; Ate, Atezolizumab; Tis, Tislelizumab; AVE, Avelumab; Niv, Nivolumab; Pem, Pembrolizumab; Dur, Durvalumab; Cem, Cemiplimab; Bev,Bevacizumab; CBP, Carboplatin

First author, year	Treatment	Any Event（1-5）	Any Event（3-5）	Pneumonitis（1-5）	Pneumonitis（3-5）	Colitis（1-5）	Colitis（3-5）	Hypothyroidism（1-5）	Hypothyroidism（3-5）	Hyperthyroidism（1-5）	Hyperthyroidism（3-5）	Hepatitis（1-5）	Hepatitis（3-5）	Rash（1-5）	Rash（3-5）
Socinski MA, 2018	Ate+Bev+PTX + CBP	280	51	11	6	9	5	50	1	16	1	55	16	113	9
	Bev+PTX+CBP	115	10	5	4	2	2	15	0	5	0	20	3	52	2
Sugawara S, 2021	Niv+Bev+PTX + CBP	226	67	20	7	17	10	28	1	15	0	4	3	112	34
	Placebo+Bev+PTX + CBP	73	9	3	2	2	1	7	0	4	0	1	1	47	4
West H, 2019	Ate+Nab-PTX+CBP	238	40	31	0	5	5	70	3	23	1	46	c		
	nab-PTX+CBP	50	17	3	1	1	0	1	0	1	0	19	7		
Jotte R, 2020	Ate+CBP+nab-PTX	241	45	25	5	6	4	37	2	12	1	62	20	77	6
	Ate+CBP+PTX	246	39	25	10	5	2	35	0	12	0	62	13	82	6
	CBP+nab-PTX	81	8	5	3	0	0	3	0	1	0	30	4	39	1
Moore KN, 2021	Ate+PTX+CBP+Bev	469	110	12	1	21	11	166	3	51	0	17	7	265	41
	Placebo+PTX+CBP+Bev	336	38	4	0	11	7	83	1	23	0	14	4	165	6
Wang J, 2021	Tis+CBP+PTX	71	11	2	0			14	0	6	0			5	4
	Tis+CBP+nab-PTX	65	12	3	2			15	0	1	0			4	2
	CBP+PTX	24	3	0	0			0	0	0	0			0	0
Monk BJ, 2021	Ave+PTX+CBP	92	24	6	1	6	3					8	6	38	9
	PTX+CBP	0	0	0	0	0	0					0	0	0	0
Hellmann MD, 2018	Nivolumab			9	6			25	1					43	3
	Platinum doublet chemotherapy			3	2			0	0					29	0
Herbst RS, 2020	Ate	148	19	11	2	3	2	27	0			46	12	44	3
	platinum-based chemotherapy	48	3	1	0	0	0	4	0			22	1	19	2
Powles T, 2021	Pem	78	19	13	4	5	2	31	1	10	0	2	1	40	0
	Standard-of-care Chemotherapy	8	4	2	1	2	1	1	0	0	0	0	0	24	2
Powles T, 2020	Dur			6	2	15	7	24	2	22	0	4	1	51	4
	Standard of Care Chemotherapy			1	0	0	0	0	0	0	0	0	0	12	0
Sezer A, 2021	Cem	62	13	1	1	3	0	20	0	15	0	2	1	3	2
	Standard-of-care Chemotherapy	8	1	0	0	0	0	0	0	3	0	0	0	0	0
Rizvi NA, 2020	Dur	50	16	8	5	2	1	21	2	4	0	1	1	5	4
	Standard-of-care Chemotherapy	12	3	5	2	0	0	2	0	1	0	0	0	2	0
Shitara K, 2020	Pem	54	15	9		7		21		11		3			
	Placebo +Standard-of-care Chemotherapy	19	4	1		1		10		0		1			
Reck M, 2021	Pem	53	21	13	5	6	3	16	0	11	0	2	2		
	platinum-based chemotherapy	8	1	1	1	0	0	3	0	2	0	0	0		
Mok TSK, 2019	Pem	177	51	53	22	7	4	77	1	39	1	9	7		
	platinum-based chemotherapy	44	9	3	1	2	1	9	0	4	0	0	0		

CarbEone	Niv			7	4			17	0					26	2
DP, 2017	platinum-based chemotherapy			0	0			1	0					15	1
Emens LA,	Ate plus nab-PTX	270	40	18	2	7	2	84	0	22	1	11	7	165	5
2021	nab-PTX+ placebo	179	21	1	0	3	1	19	0	5	0	7	1	112	2
Miles D,	Ate+PTX	268	49	16	3	3	1	60	0	25	0	7	2	141	4
2021	placebo+PTX	116	11	3	0	2	2	12	0	0	0	2	0	66	2
Schmid P,	Pem+ PTX+ CBP	304	101	10	3	13	7	107	3	36	2	11	9		
2020	Placebo+ PTX+CBP	71	7	5	1	3	1	13	0	4	0	2	0		
Shitara K,	Pem	61	10	8	2	3	1	23	0	12	0	4	4		
2018	PTX	21	5	0	0	4	3	1	0	1	0	0	0		
Bellmunt J,	Pem	45	12	11	6	6	3	17	0	10	0				
2017	Chemotherapy	19	4	1	0	1	0	3	0	1	0				

Supplementary Table 2. The occurrence of irAEs in different treatment groups

NSCLC, non-small cell lung cancer; PTX, Paclitaxel; nab-PTX, nanoparticle albumin-bound paclitaxel; Ate, Atezolizumab; Tis, Tislelizumab; AVE, Avelumab; Niv, Nivolumab; Pem, Pembrolizumab; Dur, Durvalumab; Cem, Cemiplimab; Bev,Bevacizumab; CBP, Carboplatin.

Grade 3-5 Pneumonitis					
chemotherapy	**6.47 (2.77,15.14)**	**3.66 (1.11,12.06)**	2.13 (0.70,6.55)	**3.51 (1.48,8.30)**	1.92 (0.71,5.18)
**0.11 (0.05,0.21)**	PD-1	0.56 (0.13,2.45)	0.33 (0.08,1.35)	0.54 (0.16,1.82)	0.30 (0.08,1.10)
**0.28 (0.11,0.75)**	2.66 (0.81,8.76)	PD-L1	0.58 (0.11,3.00)	0.96 (0.22,4.18)	0.53 (0.11,2.48)
**0.33 (0.15,0.76)**	**3.15 (1.09,9.14)**	1.18 (0.34,4.11)	PD-1+PTX	1.64 (0.41,6.54)	0.90 (0.22,3.72)
**0.28 (0.15,0.51)**	**2.65 (1.08,6.52)**	1.00 (0.31,3.19)	0.84 (0.31,2.30)	PD-L1+PTX	0.55 (0.21,1.43)
**0.20 (0.10,0.41)**	1.93 (0.72,5.13)	0.72 (0.23,2.32)	0.61 (0.23,1.61)	0.73 (0.34,1.57)	ICI+nab-PTX
Grade 1-5 Pneumonitis					

Grade 3-5 Colitis					
chemotherapy	2.44 (0.85,7.01)	2.64 (0.39,18.02)	**6.30 (1.69,23.54)**	1.55 (0.74,3.21)	3.19 (0.95,10.69)
**0.33 (0.16,0.69)**	PD-1	1.08 (0.12,9.65)	2.58 (0.48,13.93)	0.63 (0.18,2.28)	1.31 (0.26,6.49)
**0.17 (0.03,0.96)**	0.51 (0.08,3.33)	PD-L1	2.39 (0.23,24.59)	0.59 (0.07,4.58)	1.21 (0.13,11.73)
**0.22 (0.09,0.55)**	0.67 (0.21,2.14)	1.31 (0.19,9.19)	PD-1+PTX	0.25 (0.05,1.11)	0.51 (0.08,3.03)
**0.48 (0.27,0.85)**	1.43 (0.57,3.62)	2.80 (0.45,17.29)	2.14 (0.73,6.29)	PD-L1+PTX	2.07 (0.62,6.88)
**0.39 (0.17,0.91)**	1.18 (0.39,3.59)	2.30 (0.34,15.73)	1.76 (0.51,6.10)	0.82 (0.35,1.94)	ICI+nab-PTX
Grade 1-5 Colitis					

Grade 3-5 Hepatitis					
chemotherapy	**6.02 (1.45,25.01)**	**5.85 (1.14,30.01)**	**6.44 (1.28,32.33)**	**2.58 (1.14,5.84)**	**2.73 (1.18,6.33)**
**0.17 (0.05,0.54)**	PD-1	0.97 (0.11,8.51)	1.07 (0.12,9.22)	0.43 (0.08,2.22)	0.45 (0.09,2.38)
**0.44 (0.22,0.86)**	2.60 (0.68,10.02)	PD-L1	1.10 (0.11,10.96)	0.44 (0.07,2.76)	0.47 (0.07,2.95)
**0.24 (0.07,0.78)**	1.42 (0.27,7.45)	0.55 (0.14,2.12)	PD-1+PTX	0.40 (0.07,2.44)	0.42 (0.07,2.61)
**0.50 (0.34,0.74)**	2.96 (0.87,10.10)	1.14 (0.52,2.50)	2.08 (0.60,7.23)	PD-L1+PTX	1.06 (0.41,2.72)
**0.59 (0.38,0.90)**	**3.50 (1.01,12.08)**	1.34 (0.60,3.02)	2.46 (0.70,8.65)	1.18 (0.73,1.91)	ICI+nab-PTX
Grade 1-5 Hepatitis					

Grade 3-5 Rash					
chemotherapy	7.12 (0.85,59.49)	2.45 (0.75,8.06)	**9.63 (4.12,22.51)**	**4.70 (2.53,8.73)**	**4.21 (1.78,9.94)**
**0.41 (0.21,0.81)**	PD-1	0.34 (0.03,3.93)	1.35 (0.14,13.30)	0.66 (0.07,6.02)	0.59 (0.06,5.84)
**0.50 (0.32,0.79)**	1.23 (0.55,2.76)	PD-L1	3.92 (0.91,16.91)	1.91 (0.50,7.32)	1.71 (0.40,7.44)
**0.27 (0.15,0.48)**	0.66 (0.27,1.58)	0.54 (0.26,1.11)	PD-1+PTX	0.49 (0.18,1.34)	0.44 (0.15,1.24)
**0.52 (0.39,0.69)**	1.26 (0.60,2.62)	1.03 (0.60,1.75)	**1.91 (1.02,3.58)**	PD-L1+PTX	0.90 (0.37,2.15)
**0.53 (0.36,0.79)**	1.30 (0.60,2.82)	1.06 (0.58,1.92)	**1.97 (1.04,3.75)**	1.03 (0.67,1.59)	ICI+nab-PTX
Grade 1-5 Rash					

Grade 1-5 Hyperthyroidism					
chemotherapy	**8.52 (4.65,15.61)**	**6.33 (2.03,19.72)**	**5.45 (2.73,10.89)**	**2.92 (1.94,4.41)**	**3.60 (1.98,6.53)**
**0.13 (0.07,0.24)**	PD-1	0.74 (0.21,2.69)	0.64 (0.26,1.61)	**0.34 (0.17,0.71)**	**0.42 (0.18,0.99)**
**0.09 (0.04,0.24)**	0.72 (0.24,2.18)	PD-L1	0.86 (0.23,3.25)	0.46 (0.14,1.54)	0.57 (0.16,2.05)
**0.20 (0.10,0.40)**	1.52 (0.60,3.82)	2.11 (0.66,6.80)	PD-1+PTX	0.54 (0.24,1.18)	0.66 (0.28,1.56)
**0.27 (0.16,0.48)**	2.11 (0.95,4.72)	**2.94 (1.00,8.63)**	1.39 (0.59,3.28)	PD-L1+PTX	1.23 (0.67,2.26)
**0.16 (0.08,0.33)**	1.23 (0.52,2.95)	1.72 (0.54,5.42)	0.81 (0.35,1.87)	0.58 (0.28,1.23)	ICI+nab-PTX
Grade 1-5 Hypothyroidism					

Grade 1-5 Any Event					
chemotherapy	**5.31 (3.12,9.04)**	**4.63 (1.76,12.20)**	**7.04 (3.38,14.66)**	**3.54 (1.88,6.66)**	**3.89 (2.08,7.30)**
**0.21 (0.12,0.37)**	PD-1	0.87 (0.29,2.64)	1.33 (0.54,3.27)	0.67 (0.29,1.52)	0.73 (0.32,1.67)
**0.18 (0.06,0.51)**	0.83 (0.25,2.77)	PD-L1	1.52 (0.45,5.12)	0.76 (0.24,2.43)	0.84 (0.26,2.67)
**0.15 (0.08,0.28)**	0.70 (0.29,1.65)	0.84 (0.24,2.95)	PD-1+PTX	0.50 (0.19,1.31)	0.55 (0.23,1.34)
**0.29 (0.17,0.49)**	1.37 (0.64,2.93)	1.66 (0.51,5.43)	1.97 (0.87,4.44)	PD-L1+PTX	1.10 (0.48,2.50)
**0.34 (0.20,0.59)**	1.63 (0.75,3.51)	1.97 (0.60,6.49)	**2.33 (1.09,5.00)**	1.19 (0.62,2.28)	ICI+nab-PTX
Grade 3-5 Any Event					

Supplementary Table 3. The odds ratios (ORs) for pairwise comparisons of subgroup irAEs based on network consistency model. This is an indirect comparison of adverse events of grades 1-5 and 3-5 in different treatment regimens. The combined odds ratios and 95% confidence intervals indicate the results between the highest and

lowest treatment regimens. Each unit contains the combined odds ratio and 95% confidence interval, with significant results highlighted in thick line.

Grade 1-5 Any Event							Grade 3-5 Any Event						
Treatment	Probability				SUCRA	MeanRank	Treatment	Probability				SUCRA	MeanRank
	Rank1	Rank2	Rank3	Rank4				Rank1	Rank2	Rank3	Rank4		
ICI+nab-PTX	13.0	23.6	63.3	0.0	49.9	2.5	ICI+nab-PTX	2.6	10.6	86.8	0.0	38.6	2.8
ICI+PTX	33.4	45.8	20.8	0.0	70.8	1.9	ICI+PTX	36.6	57.1	6.3	0.0	76.8	1.7
ICI	53.6	30.6	15.8	0.0	79.3	1.6	ICI	60.8	32.3	6.9	0.0	84.6	1.5
chemotherapy	0.0	0.0	0.0	100.0	0.0	4.0	chemotherapy	0.0	0.0	0.0	100.0	0.0	4.0

Grade 1-5 Pneumonitis							Grade 3-5 Pneumonitis						
Treatment	Probability				SUCRA	MeanRank	Treatment	Probability				SUCRA	MeanRank
	Rank1	Rank2	Rank3	Rank4				Rank1	Rank2	Rank3	Rank4		
ICI+nab-PTX	24.6	61.4	14.0	0.0	70.2	1.9	ICI+nab-PTX	1.4	19.2	58.2	21.2	33.6	3.0
ICI+PTX	1.2	15.7	83.1	0.0	39.4	2.8	ICI+PTX	2.4	77.1	20.2	0.3	60.6	2.2
ICI	74.2	22.8	3.0	0.0	90.4	1.3	ICI	96.2	3.3	0.5	0.0	98.6	1.0
chemotherapy	0.0	0.0	0.0	100.0	0.0	4.0	chemotherapy	0.0	0.4	21.0	78.6	7.3	3.8

Grade 1-5 Colitis							Grade 3-5 Colitis						
Treatment	Probability				SUCRA	MeanRank	Treatment	Probability				SUCRA	MeanRank
	Rank1	Rank2	Rank3	Rank4				Rank1	Rank2	Rank3	Rank4		
ICI+nab-PTX	31.3	32.2	35.6	1.0	64.6	2.1	ICI+nab-PTX	69.0	19.6	10.1	1.4	85.4	1.4
ICI+PTX	14.9	41.3	43.8	0.0	57.0	2.3	ICI+PTX	9.9	45.3	44.0	0.8	54.8	2.4
ICI	53.9	26.6	19.5	0.0	78.1	1.7	ICI	21.1	34.9	39.0	5.0	57.3	2.3
chemotherapy	0.0	0.0	1.0	99.0	0.3	4.0	chemotherapy	0.0	0.2	7.0	92.8	2.5	3.9

Grade 1-5 Hepatitis							Grade 3-5 Hepatitis						
Treatment	Probability				SUCRA	MeanRank	Treatment	Probability				SUCRA	MeanRank
	Rank1	Rank2	Rank3	Rank4				Rank1	Rank2	Rank3	Rank4		
ICI+nab-PTX	5.9	25.9	68.0	0.2	45.8	2.6	ICI+nab-PTX	8.8	37.1	53.4	0.6	51.4	2.5
ICI+PTX	12.7	63.4	23.9	0.0	62.9	2.1	ICI+PTX	11.6	50.6	37.7	0.1	57.9	2.3
ICI	81.4	10.6	7.9	0.0	91.2	1.3	ICI	79.5	12.3	8.2	0.0	90.4	1.3
chemotherapy	0.0	0.0	0.2	99.8	0.1	4.0	chemotherapy	0.0	0.0	0.7	99.3	0.2	4.0

Grade 1-5 Rash							Grade 3-5 Rash						
Treatment	Probability				SUCRA	MeanRank	Treatment	Probability				SUCRA	MeanRank
	Rank1	Rank2	Rank3	Rank4				Rank1	Rank2	Rank3	Rank4		
ICI+nab-PTX	14.7	27.3	57.9	0.1	52.2	2.4	ICI+nab-PTX	17.2	50.0	32.7	0.1	61.5	2.2
ICI+PTX	47.4	40.0	32.7	0.0	78.3	1.7	ICI+PTX	69.8	27.0	3.2	0.0	88.9	1.3
ICI	37.9	32.7	29.4	0.0	69.5	1.9	ICI	13.0	23.0	62.5	1.5	49.2	2.5
chemotherapy	0.0	0.0	0.1	99.9	0.0	4.0	chemotherapy	0.0	0.0	1.6	98.4	0.5	4.0

Grade 1-5 Hypothyroidism							Grade 1-5 Hyperthyroidism						
Treatment	Probability				SUCRA	MeanRank	Treatment	Probability				SUCRA	MeanRank
	Rank1	Rank2	Rank3	Rank4				Rank1	Rank2	Rank3	Rank4		
ICI+nab-PTX	20.0	69.4	10.7	0.0	69.8	1.9	ICI+nab-PTX	4.1	52.6	43.3	0.0	53.6	2.4
ICI+PTX	0.5	11.5	88.1	0.0	37.5	2.9	ICI+PTX	1.5	42.8	55.7	0.0	48.6	2.5
ICI	79.6	19.1	1.3	0.0	92.8	1.2	ICI	94.4	4.7	0.9	0.0	97.8	1.1
chemotherapy	0.0	0.0	0.0	100.0	0.0	4.0	chemotherapy	0.0	0.0	0.0	100.0	0.0	4.0

Supplementary Table 4. Rank probabilities with surface under the cumulative ranking curve (SUCRA) and Mean Rank value for irAEs in four treatment groups based on the network consistency model. The ranking of the different approaches for all outcomes is reported. The probability of each approach being ranked from best to worst is presented in percentage form in the row. SUCRA, a higher SUCRA value indicates a higher rank or better performance of the intervention for that particular outcome, while a lower value indicates a lower rank or worse performance.

Grade 1-5 Any Event								
Treatment	Probability						SUCRA	MeanRank
	Rank1	Rank2	Rank3	Rank4	Rank5	Rank6		
ICI+nab-PTX	3.5	11.8	57.5	18.9	17.8	0.0	45.9	3.7
PD-L1+PTX	2.3	7.2	24.6	19.7	36.8	0.0	39.5	4.0
PD-1+PTX	0.9	18.0	11.6	20.1	26.6	0.0	86.3	1.7
PD-L1	4.4	20.6	4.4	18.2	13.5	0.0	58.6	3.1
PD-1	85.9	41.6	1.9	23.0	5.3	0.1	69.7	2.5
chemotherapy	0.0	0.0	0.0	0.1	0.0	99.9	0.0	6.0

Grade 3-5 Any Event								
Treatment	Probability						SUCRA	MeanRank
	Rank1	Rank2	Rank3	Rank4	Rank5	Rank6		
ICI+nab-PTX	0.2	2.3	9.6	29.0	58.9	0.0	31.2	4.4
PD-L1+PTX	1.1	6.8	23.2	44.5	24.4	0.0	43.1	3.8
PD-1+PTX	53.0	32.4	11.4	2.7	0.5	0.0	87.0	1.7
PD-L1	35.1	26.6	17.6	10.1	10.6	0.0	73.0	2.3
PD-1	10.7	31.9	38.2	13.8	5.5	0.1	65.7	2.7
chemotherapy	0.0	0.0	0.0	0.1	0.0	99.9	0.0	6.0

Grade 1-5 Pneumonitis								
Treatment	Probability						SUCRA	MeanRank
	Rank1	Rank2	Rank3	Rank4	Rank5	Rank6		
ICI+nab-PTX	8.0	46.7	29.1	12.5	3.8	0.0	68.5	2.6
PD-L1+PTX	0.7	12.7	30.9	33.9	21.8	0.0	47.4	3.6
PD-1+PTX	0.9	9.0	17.6	28.7	43.3	0.5	38.8	4.1
PD-L1	4.4	20.6	20.1	24.4	29.7	0.8	48.7	3.6
PD-1	85.9	11.0	2.3	0.6	0.1	0.0	96.4	1.2
chemotherapy	0.0	0.0	0.0	0.0	1.3	98.7	0.3	6.0

Grade 3-5 Pneumonitis								
Treatment	Probability						SUCRA	MeanRank
	Rank1	Rank2	Rank3	Rank4	Rank5	Rank6		
ICI+nab-PTX	1.0	4.1	14.6	33.3	38.2	8.7	34.1	4.3
PD-L1+PTX	10.1	33.1	35.9	17.4	3.4	0.1	65.7	2.7
PD-1+PTX	3.4	10.3	19.0	29.2	30.0	8.1	40.7	4.0
PD-L1	18.4	30.1	23.0	16.1	10.8	1.6	64.8	2.8
PD-1	67.1	22.4	7.4	2.5	0.6	0.0	90.6	1.5
chemotherapy	0.0	0.0	0.1	1.5	17.0	81.5	4.0	5.8

Grade 1-5 Colitis								
Treatment	Probability						SUCRA	MeanRank
	Rank1	Rank2	Rank3	Rank4	Rank5	Rank6		
ICI+nab-PTX	4.9	14.5	25.4	30.2	23.5	1.5	48.5	3.6
PD-L1+PTX	0.4	3.7	14.6	34.8	45.8	0.6	35.2	4.2
PD-1+PTX	32.2	40.4	16.2	7.5	3.7	0.1	77.9	2.1
PD-L1	55.6	17.7	9.7	6.4	8.2	2.4	79.8	2.0
PD-1	7.0	23.8	34.1	21.0	14.0	0.1	57.7	3.1
chemotherapy	0.0	0.0	0.0	0.1	4.7	95.2	1.0	6.0

Grade 3-5 Colitis								
Treatment	Probability						SUCRA	MeanRank
	Rank1	Rank2	Rank3	Rank4	Rank5	Rank6		
ICI+nab-PTX	15.2	31.5	28.3	16.9	5.8	2.4	65.2	2.7
PD-L1+PTX	0.2	2.2	13.3	33.5	40.9	9.9	31.5	4.4
PD-1+PTX	60.2	24.6	9.7	3.9	1.3	0.2	87.6	1.6
PD-L1	18.2	20.2	18.9	15.6	11.2	15.8	54.2	3.3

PD-1	6.2	21.5	29.5	25.9	12.7	4.2	54.0	3.3
chemotherapy	0.0	0.0	0.3	4.2	28.1	67.4	7.5	5.6

Grade 1-5 Hepatitis								
Treatment	Probability						SUCRA	MeanRank
	Rank1	Rank2	Rank3	Rank4	Rank5	Rank6		
ICI+nab-PTX	0.2	1.9	11.3	30.3	55.6	0.7	31.7	4.4
PD-L1+PTX	0.3	6.4	32.2	44.2	16.9	0.0	45.8	3.7
PD-1+PTX	32.6	46.9	10.1	4.6	4.8	1.0	79.0	2.1
PD-L1	2.4	17.6	41.6	18.8	18.5	1.1	52.7	3.4
PD-1	64.6	27.1	4.8	2.1	1.3	0.1	90.2	1.5
chemotherapy	0.0	0.0	0.0	0.0	2.9	97.1	0.6	6.0

Grade 3-5 Hepatitis								
Treatment	Probability						SUCRA	MeanRank
	Rank1	Rank2	Rank3	Rank4	Rank5	Rank6		
ICI+nab-PTX	1.7	7.9	21.1	37.7	30.7	0.9	41.9	3.9
PD-L1+PTX	1.0	5.9	19.4	35.6	37.0	1.2	39.0	4.1
PD-1+PTX	35.6	28.4	18.6	8.3	7.9	1.2	74.4	2.3
PD-L1	30.4	27.2	20.3	9.5	10.9	1.8	70.3	2.5
PD-1	31.3	30.7	20.7	8.7	8.0	0.7	73.3	2.3
chemotherapy	0.0	0.0	00	0.2	5.6	94.2	1.2	5.9

Grade 1-5 Rash								
Treatment	Probability						SUCRA	MeanRank
	Rank1	Rank2	Rank3	Rank4	Rank5	Rank6		
ICI+nab-PTX	0.6	11.3	23.6	29.8	34.6	0.1	42.7	3.9
PD-L1+PTX	0.5	10.6	30.8	35.8	22.3	0.0	46.2	3.7
PD-1+PTX	80.1	16.0	2.6	0.9	0.4	0.0	94.9	1.3
PD-L1	2.4	19.5	27.5	22.5	27.9	0.2	49.1	3.5
PD-1	16.4	42.7	15.4	11.0	14.0	0.5	67.0	2.7
chemotherapy	0.0	0.0	0.0	0.0	0.8	99.2	0.2	6.0

Grade 3-5 Rash								
Treatment	Probability						SUCRA	MeanRank
	Rank1	Rank2	Rank3	Rank4	Rank5	Rank6		
ICI+nab-PTX	2.5	15.5	31.4	35.8	14.8	0.1	51.0	3.5
PD-L1+PTX	2.9	20.7	42.1	28.3	6.1	0.0	57.2	3.1
PD-1+PTX	53.5	37.9	6.5	1.8	0.4	0.0	88.5	1.6
PD-L1	1.4	5.6	10.2	19.6	56.0	7.2	31.0	4.4
PD-1	39.8	20.4	9.7	14.3	12.3	3.5	70.2	2.5
chemotherapy	0.0	0.0	0.0	7.2	3.5	89.3	2.2	5.9

Grade 1-5 Hypothyroidism								
Treatment	Probability						SUCRA	MeanRank
	Rank1	Rank2	Rank3	Rank4	Rank5	Rank6		
ICI+nab-PTX	9.1	24.4	39.1	22.7	4.7	0.0	62.1	2.9
PD-L1+PTX	0.1	1.0	5.7	21.2	72.0	0.0	27.2	4.6
PD-1+PTX	3.5	11.7	25.0	40.1	19.7	0.0	47.9	3.6
PD-L1	64.7	18.8	9.2	5.4	1.8	0.0	87.8	1.6
PD-1	22.5	44.1	21.0	10.5	1.8	0.0	75.0	2.2
chemotherapy	0.0	0.0	0.0	0.0	0.0	100.0	0.0	6.0

Grade 1-5 Hyperthyroidism								
Treatment	Probability						SUCRA	MeanRank
	Rank1	Rank2	Rank3	Rank4	Rank5	Rank6		
ICI+nab-PTX	0.8	5.7	22.0	49.2	22.3	0.0	42.7	3.9

PD-L1+PTX	0.0	0.5	5.8	29.5	64.1	0.0	28.5	4.6
PD-1+PTX	10.7	33.7	40.9	10.9	3.8	0.0	67.3	2.6
PD-L1	29.2	29.3	22.4	9.4	9.5	0.1	71.8	2.4
PD-1	59.3	30.8	8.8	0.9	0.1	0.0	89.6	1.5
chemotherapy	0.0	0.0	0.0	0.0	0.1	99.9	0.0	6.0

Supplementary Table 5. Rank probabilities with surface under the cumulative ranking curve (SUCRA) and Mean Rank value for irAEs in six treatment groups based on the network consistency model. The ranking of the different approaches for all outcomes is reported. The probability of each approach being ranked from best to worst is presented in percentage form in the row. SUCRA, a higher SUCRA value indicates a higher rank or better performance of the intervention for that particular outcome, while a lower value indicates a lower rank or worse performance.

Outcomes	Loop	IF	seIF	CI_95	P Value
**four treatment groups**					
Grade 1-5 Any Event	ICI+nab-PTX-ICI+PTX-chemotherapy	0.055	0.899	(0.00,1.82)	0.951
Grade 3-5 Any Event	ICI+nab-PTX-ICI+PTX-chemotherapy	0.890	0.654	(0.00,2.17)	0.174
Grade 1-5 Pneumonitis	ICI+nab-PTX-ICI+PTX-chemotherapy	0.821	0.516	(0.00,1.83)	0.112
Grade 3-5 Pneumonitis	ICI+nab-PTX-ICI+PTX-chemotherapy	0.326	0.855	(0.00,2.00)	0.703
Grade 1-5 Colitis	ICI+nab-PTX-ICI+PTX-chemotherapy	0.082	0.951	(0.00,1.95)	0.931
Grade 3-5 Colitis	ICI+nab-PTX-ICI+PTX-chemotherapy	0.034	1.211	(0.00,2.41)	0.977
Grade 1-5 Hepatitis	ICI+nab-PTX-ICI+PTX-chemotherapy	0.110	0.375	(0.00,0.84)	0.768
Grade 3-5 Hepatitis	ICI+nab-PTX-ICI+PTX-chemotherapy	0.924	0.948	(0.00,2.78)	0.330
Grade 1-5 Rash	ICI+nab-PTX-ICI+PTX-chemotherapy	0.015	0.673	(0.00,1.34)	0.982
Grade 3-5 Rash	ICI+nab-PTX-ICI+PTX-chemotherapy	0.335	0.823	(0.00,1.95)	0.684
Grade 1-5 Hypothyroidism	ICI+nab-PTX-ICI+PTX-chemotherapy	0.835	0.467	(0.00,1.75)	0.074
Grade 1-5 Hyperthyroidism	ICI+nab-PTX-ICI+PTX-chemotherapy	0.906	0.587	(0.00,2.06)	0.123
**six treatment groups**					
Grade 1-5 Any Event	ICI+nab-PTX-PD-L1 +PTX-chemotherapy	0.035	0.892	(0.00,1.78)	0.456
	ICI+nab-PTX-PD-1 +PTX-chemotherapy	0.555	1.371	(0.00,3.24)	0.653
Grade 3-5 Any Event	ICI+nab-PTX-PD-L1 +PTX-chemotherapy	0.949	0.502	(0.00,1.93)	0.059
	ICI+nab-PTX-PD-1 +PTX-chemotherapy	1.411	0.964	(0.00,3.30)	0.143
Grade 1-5 Pneumonitis	ICI+nab-PTX-PD-L1 +PTX-chemotherapy	0.801	0.640	(0.00,2.06)	0.211
	ICI+nab-PTX-PD-1 +PTX-chemotherapy	0.426	1.270	(0.00,2.92)	0.737
Grade 3-5 Pneumonitis	ICI+nab-PTX-PD-L1 +PTX-chemotherapy	0.065	1.164	(0.00,2.35)	0.956
	ICI+nab-PTX-PD-1 +PTX-chemotherapy	2.120	1.766	(0.00,5.58)	0.230
Grade 1-5 Colitis	ICI+nab-PTX-PD-L1 +PTX-chemotherapy	0.207	0.860	(0.00,1.89)	0.810
Grade 3-5 Colitis	ICI+nab-PTX-PD-L1 +PTX-chemotherapy	0.432	1.193	(0.00,2.77)	0.717
Grade 1-5 Hepatitis	ICI+nab-PTX-PD-L1 +PTX-chemotherapy	0.190	0.545	(0.00,1.26)	0.727
Grade 3-5 Hepatitis	ICI+nab-PTX-PD-L1 +PTX-chemotherapy	0.570	1.165	(0.00,2.85)	0.624
Grade 1-5 Rash	ICI+nab-PTX-PD-L1 +PTX-chemotherapy	0.162	0.528	(0.00,1.20)	0.759
	ICI+nab-PTX-PD-1 +PTX-chemotherapy	0.604	1.300	(0.00,3.15)	0.642
Grade 3-5 Rash	ICI+nab-PTX-PD-L1 +PTX-chemotherapy	0.180	1.021	(0.00,2.18)	0.860
	ICI+nab-PTX-PD-1 +PTX-chemotherapy	0.477	1.125	(0.00,2.68)	0.671
Grade 1-5 Hypothyroidism	ICI+nab-PTX-PD-L1 +PTX-chemotherapy	0.833	0.960	(0.00,2.72)	0.386
	ICI+nab-PTX-PD-1 +PTX-chemotherapy	0.450	0.971	(0.00,2.35)	0.642
Grade 1-5 Hyperthyroidism	ICI+nab-PTX-PD-L1 +PTX-chemotherapy	0.775	0.620	(0.00,1.99)	0.212
	ICI+nab-PTX-PD-1 +PTX-chemotherapy	1.749	1.072	(0.00,3.85)	0.103

Supplementary Table 6. Evaluation of inconsistency using loop-specific approach heterogeneity estimates. "IF" refers to the estimated inconsistency factor for a particular loop in the network, which represents the extent of inconsistency observed in that loop. "seIF" refers to the standard error of the inconsistency factor estimate. "CI_95" refers to the 95% confidence interval of the inconsistency factor estimate. "P Value" refers to the statistical significance of the inconsistency, with a significant P value indicating that the observed inconsistency is unlikely to be due to chance alone. If the P value in the loop-specific approach is greater than 0.5, it suggests that there is no statistically significant inconsistency in the loop being evaluated.

Outcomes	Comparators	Intervention	Odd Ratio (95% CrI)			P
			Direct	Indirect	Network	
**four treatment groups**						
Grade 1-5 Any Event	ICI+PTX	ICI+nab-PTX	-0.14(-0.51,1.22)	-0.84(-0.24,0.64)	-0.36(-1.38,0.62)	0.46
Grade 3-5 Any Event	ICI+PTX	ICI+nab-PTX	0.12(-0.67,0.90)	-1.31(-2.22,-0.48)	-0.50(-1.29,0.27)	0.02
**four treatment groups**						
Grade 1-5 Pneumonitis	ICI+PTX	ICI+nab-PTX	0.92(0.26,2.9)	0.34(0.062,1.6)	0.66(0.24,1.5)	0.29
Grade 3-5 Pneumonitis	ICI+PTX	ICI+nab-PTX	0.91(0.26,2.9)	0.34(0.062,1.6)	0.65(0.24,1.5)	0.29
Grade 1-5 Colitis	ICI+PTX	ICI+nab-PTX	0.83(0.085,7.7)	1.4(0.17,11)	0.95(0.21,4.1)	0.71
Grade 3-5 Colitis	ICI+PTX	ICI+nab-PTX	0.44(0.016,11)	0.38(0.0061,8.9)	0.36(0.032,3)	0.93
Grade 1-5 Hepatitis	ICI+PTX	ICI+nab-PTX	0.01(-1.83,1.85)	-0.58(-2.39,0.81)	-0.26(-1.53,0.71)	0.45
Grade 3-5 Hepatitis	ICI+PTX	ICI+nab-PTX	0.63(0.066,6.2)	4.1(0.34,69)	1.2(0.27,6.9)	0.17
Grade 1-5 Rash	ICI+PTX	ICI+nab-PTX	1.2(0.23,5.9)	2.0(0.23,26)	1.3(0.46,3.9)	0.61
Grade 3-5 Rash	ICI+PTX	ICI+nab-PTX	1.3(0.23,9.1)	2.7(0.12,52)	1.5(0.43,5.4)	0.64
Grade 1-5 Hypothyroidism	ICI+PTX	ICI+nab-PTX	0.93(0.27,3.2)	0.35(0.061,1.5)	0.55(0.18,1.5)	0.26
Grade 1-5 Hyperthyroidism	ICI+PTX	ICI+nab-PTX	1.6(0.57,6.4)	0.63（0.15，2.7）	1.1（0.45，2.9）	0.25
**six treatment groups**						
Grade 1-5 Any Event	PD-1+PTX	ICI+nab-PTX	0.20(-1.74,2.14)	1.30(-0.15,2.93)	0.91(-0.27,2.21)	0.34
Grade 3-5 Any Event	PD-1+PTX	ICI+nab-PTX	-0.05(-1.39,1.27)	1.52(0.52,2.62)	0.98(0.09,1,95)	0.06
Grade 1-5 Pneumonitis	PD-L1+PTX	ICI+nab-PTX	1.0(0.22,4.5)	0.29(0.052,1.4)	0.64(0.20,1.5)	0.21
	PD-1+PTX	ICI+nab-PTX	0.60(0.045,6.4)	0.69(0.15,3.1)	0.69(0.20,2.3)	0.92
Grade 3-5 Pneumonitis	PD-L1+PTX	ICI+nab-PTX	2.1（0.14，34）	0.70（0.010，46）	1.7（0.29，10）	0.60
	PD-1+PTX	ICI+nab-PTX	6.5e-12(9.3e-40,0.14)	2（0.20，25）	0.73（0.067，5.5）	1
Grade 1-5 Colitis	PD-L1+PTX	ICI+nab-PTX	0.82（0.092，7.2）	0.85（0.086，6.9）	0.75（0.16，3.3）	0.98
Grade 3-5 Colitis	PD-L1+PTX	ICI+nab-PTX	0.44（0.019，8.9）	0.17（0.0023，3.5）	0.26（0.024，1.8）	0.64
Grade 1-5 Hepatitis	PD-L1+PTX	ICI+nab-PTX	1.0（0.21，4.7）	1.3（0.28，5.8）	1.1（0.56，2.2）	0.69
Grade 3-5 Hepatitis	PD-L1+PTX	ICI+nab-PTX	0.64（0.028，14）	2.2（0.089，52）	1.0（0.18，7.0）	0.47
Grade 1-5 Rash	PD-L1+PTX	ICI+nab-PTX	1.1（0.10，12）	0.60（0.033，3.8）	0.90（0.25，2.3）	0.62
	PD-1+PTX	ICI+nab-PTX	1.3（0.16，11）	4.5（1.2，40）	3.1（0.98，13）	0.28
Grade 3-5 Rash	PD-L1+PTX	ICI+nab-PTX	1.0（0.070，14）	0.96（0.035，10）	1.0（0.21，3.7）	0.98
	PD-1+PTX	ICI+nab-PTX	2.2（0.17，39）	4.0（0.44，86）	3（0.67，86）	0.72
Grade 1-5 Hypothyroidism	PD-L1+PTX	ICI+nab-PTX	0.94（0.10，8.6）	0.25（0.030，1.3）	0.44（0.098，1.6）	0.28
	PD-1+PTX	ICI+nab-PTX	0.91（0.099.8.2）	0.52（0.062，3.5）	0.70（0.15，2.9）	0.67
Grade 1-5 Hyperthyroidism	PD-L1+PTX	ICI+nab-PTX	1.0（0.15，6.7）	0.88（0.18，6.5）	0.90（0.33，3.1）	0.89
	PD-1+PTX	ICI+nab-PTX	8.6(0.83,3.4e+0.2)	0.78（0.14，3.4）	1.5（0.43，6.0）	0.08

Supplementary Table 7. Evaluation of inconsistency using node-splitting approach heterogeneity estimates. The node-splitting approach heterogeneity estimates refer to the estimation of heterogeneity (variation) between direct and indirect evidence within the network using the node-splitting method. This method involves comparing the estimates of the treatment effect obtained from the direct evidence and those obtained from the indirect evidence that pass through a common node in the network. If there is a significant difference between the two estimates, it suggests that there is inconsistency in the network. If the P value obtained from the node-splitting approach is greater than 0.05, it suggests that there is no evidence of inconsistency in the network. This means that the assumption of consistency between direct and indirect evidence is valid and the network meta-analysis results can be interpreted as reliable.

PubMed

Step	Query	Results
#1	"Immune Checkpoint Inhibitors"[MeSH Terms] OR "checkpoint inhibitors immune"[Title/Abstract] OR "immune checkpoint inhibitor"[Title/Abstract] OR "checkpoint inhibitor immune"[Title/Abstract] OR "immune checkpoint blockers"[Title/Abstract] OR "checkpoint blockers immune"[Title/Abstract] OR "immune checkpoint blockade"[Title/Abstract] OR "checkpoint blockade immune"[Title/Abstract] OR "immune checkpoint inhibition"[Title/Abstract] OR "checkpoint inhibition immune"[Title/Abstract] OR "pd l1 inhibitors"[Title/Abstract] OR "pd l1 inhibitors"[Title/Abstract] OR "pd l1 inhibitor"[Title/Abstract] OR "pd l1 inhibitor"[Title/Abstract] OR "programmed death ligand 1 inhibitors"[Title/Abstract] OR "programmed death ligand 1 inhibitors"[Title/Abstract] OR "pd 1 pd l1 blockade"[Title/Abstract] OR "blockade pd 1 pd l1"[Title/Abstract] OR "pd 1 pd l1 blockade"[Title/Abstract] OR "pd 1 inhibitors"[Title/Abstract] OR "pd 1 inhibitors"[Title/Abstract] OR "pd 1 inhibitor"[Title/Abstract] OR "inhibitor pd 1"[Title/Abstract] OR "pd 1 inhibitor"[Title/Abstract] OR "programmed cell death protein 1 inhibitor"[Title/Abstract] OR "programmed cell death protein 1 inhibitors"[Title/Abstract]	16302
#2	"randomized controlled trials as topic"[MeSH Terms] OR "randomized controlled trial"[Publication Type] OR "randomized controlled trials as topic"[MeSH Terms] OR "clinical trials randomized"[All Fields] OR "clinical trials randomised"[All Fields] OR "randomized controlled trial"[Publication Type] OR "randomized controlled trials as topic"[MeSH Terms] OR "trials randomized clinical"[All Fields] OR "trials randomised clinical"[All Fields] OR "randomized controlled trial"[Publication Type] OR "randomized controlled trials as topic"[MeSH Terms] OR "controlled clinical trials randomized"[All Fields] OR "controlled clinical trials randomised"[All Fields]	715601
#3	"animal"[All Fields] OR "rat"[All Fields] OR "mouse"[All Fields] OR "zebra"[All Fields] OR "zebra fish"[All Fields]	2962410
#4	"Review"[Title]	598921
#5	"Cells"[All Fields] OR "cell line"[All Fields] OR "In vitro"[All Fields]	5252460
#6	#1 AND #2 NOT #3 NOT #4 NOT #5	325

Embase

Step	Query	Results
#1	'immune checkpoint inhibitor'/exp	11,458
#2	'checkpoint inhibitors, immune':ab,ti	38
#3	'immune checkpoint inhibitor':ab,ti	6,775
#4	'checkpoint inhibitor, immune':ab,ti	12
#5	'immune checkpoint blockers':ab,ti	465
#6	'checkpoint blockers, immune':ab,ti	11
#7	'immune checkpoint blockade':ab,ti	6,513
#8	'checkpoint blockade, immune':ab,ti	10
#9	'immune checkpoint inhibition':ab,ti	2,125
#10	'checkpoint inhibition, immune':ab,ti	4
#11	'pd-l1 inhibitors':ab,ti	1,845
#12	'pd-l1 inhibitor':ab,ti	1,050
#13	'pd l1 inhibitor':ab,ti	1,050
#14	'programmed death-ligand 1 inhibitors':ab,ti	74
#15	'programmed death ligand 1 inhibitors':ab,ti	74
#16	'pd-1-pd-l1 blockade':ab,ti	805
#17	'blockade, pd-1-pd-l1':ab,ti	5
#18	'pd 1 pd l1 blockade':ab,ti	805
#19	'pd-1 inhibitors':ab,ti	1843
#20	'pd 1 inhibitors':ab,ti	1843
#21	'pd-1 inhibitor':ab,ti	2112
#22	'inhibitor, pd-1':ab,ti	61
#23	'pd 1 inhibitor':ab,ti	2112
#24	'programmed cell death protein 1 inhibitor':ab,ti	68
#25	'programmed cell death protein 1 inhibitors':ab,ti	35
#26	#1 OR #2 OR #3 OR #4 OR #5 OR #6 OR #7 OR #8 OR#9 OR #10 OR #11 OR #12 OR #13 OR #14 OR #15 OR#16 OR #17 OR #18 OR #19 OR #20 OR #21 OR #22 OR#23 OR #24 OR #25	28910
#27	'randomized controlled trial (topic)'/exp	224,197
#28	'clinical trials, randomized':ab,ti	203

#29	'trials, randomized clinical':ab,ti	21
#30	'controlled clinical trials, randomized':ab,ti	14
#31	#27 OR #28 OR #29 OR #30	224,325
#32	'review':ab,ti	2,372,891
#33	#26 AND #31	1,027
#34	#33 NOT #32	464

Cochrane

Step	Search	Hits
#1	(Checkpoint Inhibitors, Immune):ti,ab,kw OR (Immune Checkpoint Inhibitor):ti,ab,kw OR (Checkpoint Inhibitor, Immune):ti,ab,kw OR (Immune Checkpoint Blockers):ti,ab,kw OR (Checkpoint Blockers, Immune):ti,ab,kw	1335
#2	(Checkpoint Blockade, Immune):ti,ab,kw OR (Immune Checkpoint Inhibition):ti,ab,kw OR (Checkpoint Inhibition, Immune):ti,ab,kw OR (PD-L1 Inhibitors):ti,ab,kw OR (PD L1 Inhibitors):ti,ab,kw	1116
#3	(PD-L1 Inhibitor):ti,ab,kw OR (PD L1 Inhibitor):ti,ab,kw OR (Programmed Death-Ligand 1 Inhibitors):ti,ab,kw OR (Programmed Death Ligand 1 Inhibitors):ti,ab,kw OR (PD 1 PD L1 Blockade):ti,ab,kw	1041
#4	(PD-1 Inhibitors):ti,ab,kw OR (PD 1 Inhibitors):ti,ab,kw OR (PD 1 Inhibitors PD-1 Inhibitor):ti,ab,kw OR (Inhibitor, PD-1):ti,ab,kw OR (PD 1 Inhibitor):ti,ab,kw	5512
#5	(Programmed Cell Death Protein 1 Inhibitor):ti,ab,kw OR (Programmed Cell Death Protein 1 Inhibitors):ti,ab,kw (Word variations have been searched)	224
#7	#1 or #2 or #3 or #4 or #5 or #6	6546
#8	(Review*):ti,ab,kw OR (Animal*):ti,ab,kw OR (rat*):ti,ab,kw OR (mouse):ti,ab,kw OR (zebra):ti,ab,kw OR (zebra fish):ti,ab,kw	656709
#9	#7 not #8	2544

Web of Science

#1	TS=("Immune Checkpoint Inhibitors" OR "checkpoint inhibitors immune" OR "immune checkpoint inhibitor" OR "checkpoint inhibitor immune" OR "immune checkpoint blockers" OR "checkpoint blockers immune" OR "immune checkpoint blockade" OR "checkpoint blockade immune" OR "immune checkpoint inhibition" OR "checkpoint inhibition immune" OR "pd l1 inhibitors" OR "pd l1 inhibitors" OR "pd l1 inhibitor" OR "pd l1 inhibitor" OR "programmed death ligand 1 inhibitors" OR "programmed death ligand 1 inhibitors" OR "pd 1 pd l1 blockade" OR "blockade pd 1 pd l1" OR "pd 1 pd l1 blockade" OR "pd 1 inhibitors" OR "pd 1 inhibitors" OR "pd 1 inhibitor" OR "inhibitor pd 1" OR "pd 1 inhibitor" OR "programmed cell death protein 1 inhibitor" OR "programmed cell death protein 1 inhibitors")	35253
#2	TS=("Randomized Controlled Trials as Topic" OR "Clinical Trials, Randomized" OR "Trials, Randomized Clinical" OR "Controlled Clinical Trials, Randomized")	157740
#3	TS=("Review*")	4584211
#4	TS=("Animal*" OR "rat*" OR "mouse" OR" zebra" OR "zebra fish")	38299312
#5	TS=("Cells" OR "cell line*" OR "In vitro")	9748354
#6	#2 AND #1 NOT #5 NOT #4 NOT #3	43

Supplementary Table 8. Search strategy

1.2 Supplementary Figures

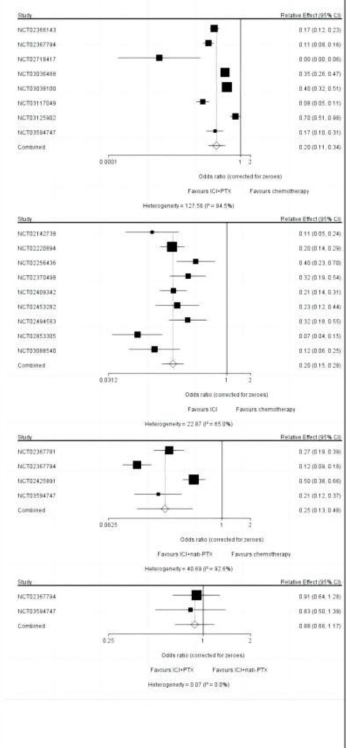

1-5 Any Event

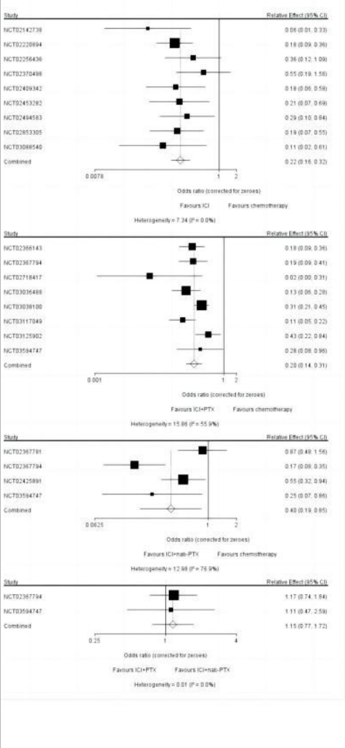

3-5 Any Event

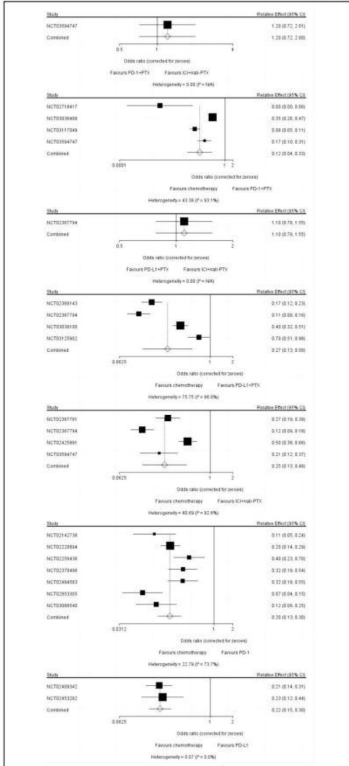

1-5 Any Event-subgroup

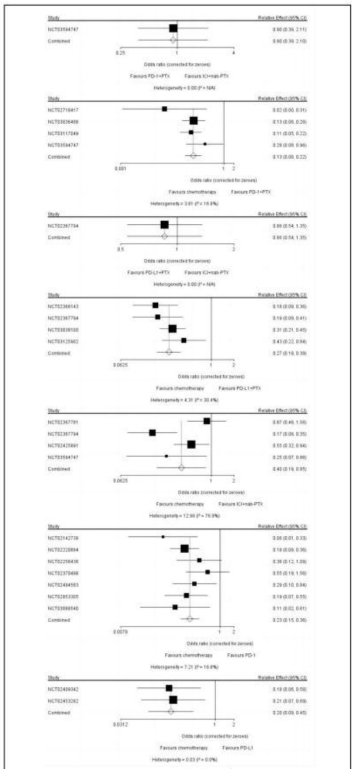

3-5 Any Event-subgroup

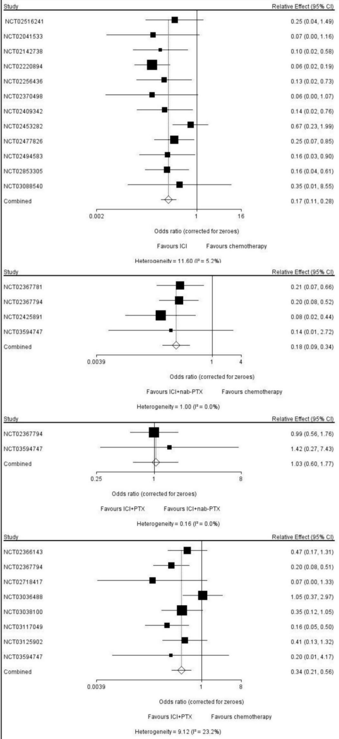

1-5 Pneumonitis

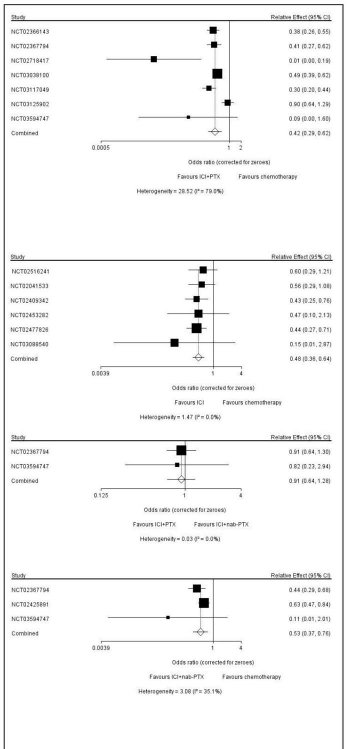

1-5 Rash

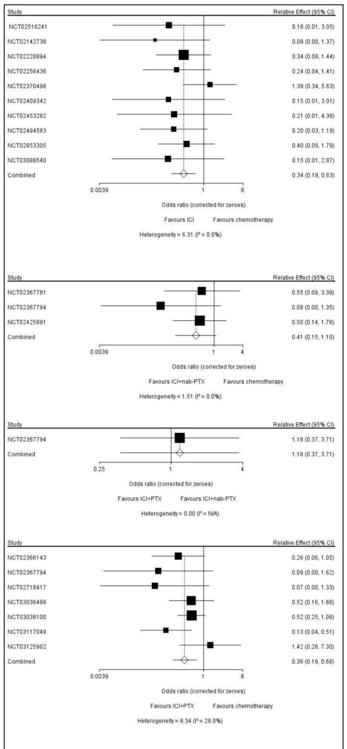

1-5 Colitis

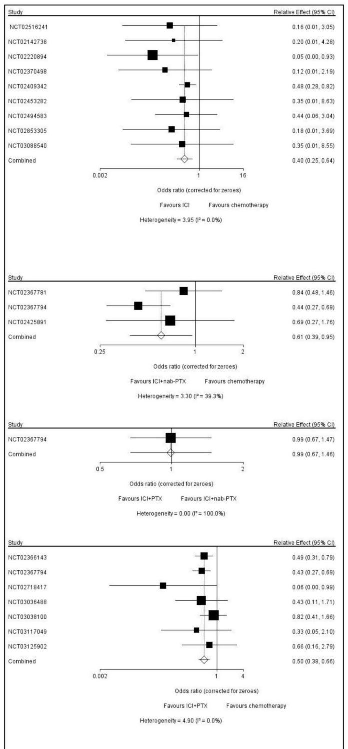

1-5 Hepatitis

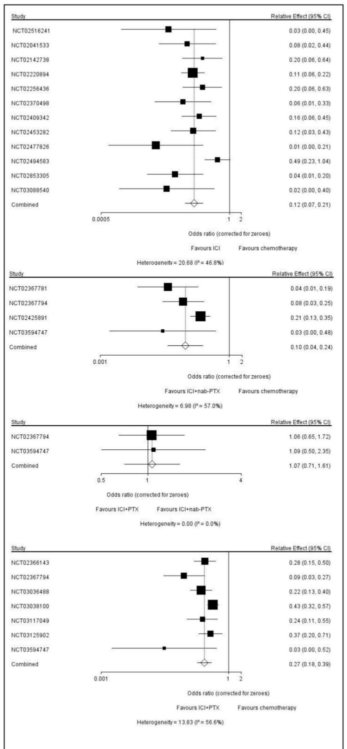

1-5 Hypothyroidism

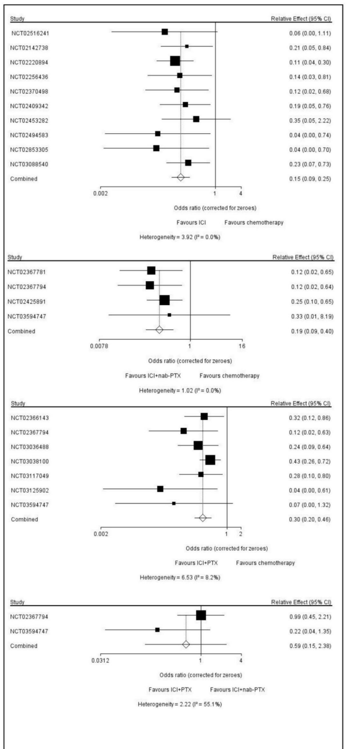

1-5 Hyperthyroidism

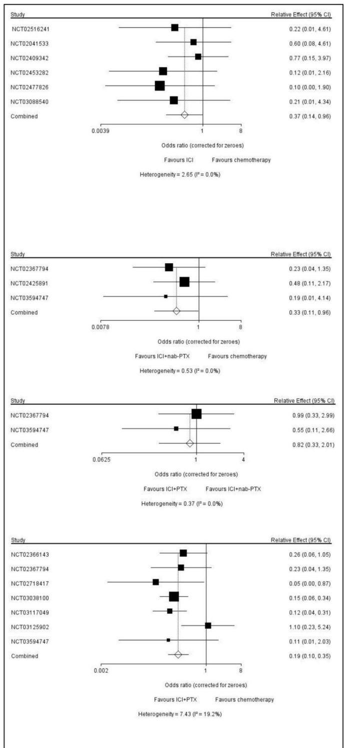

3-5 Rash

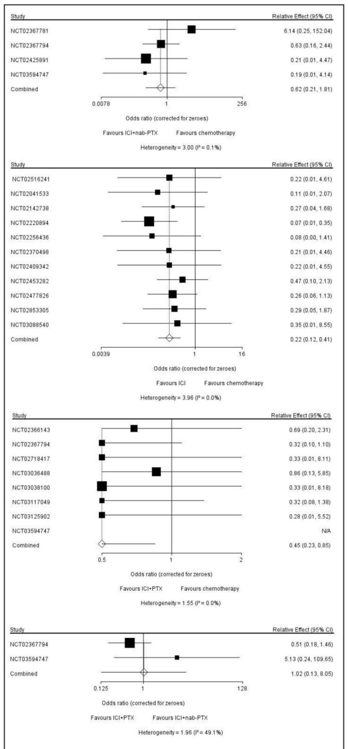

3-5 Pneumonitis

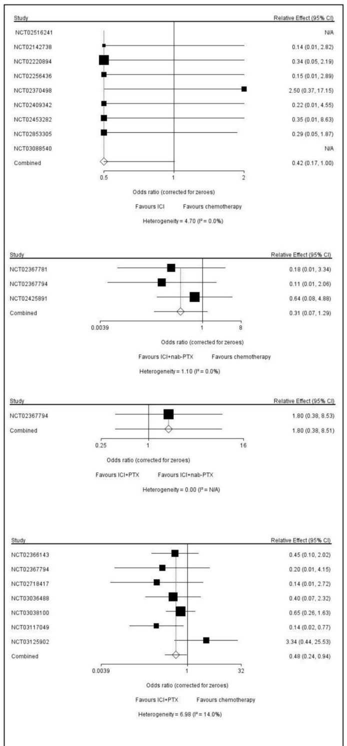

3-5 Colitis

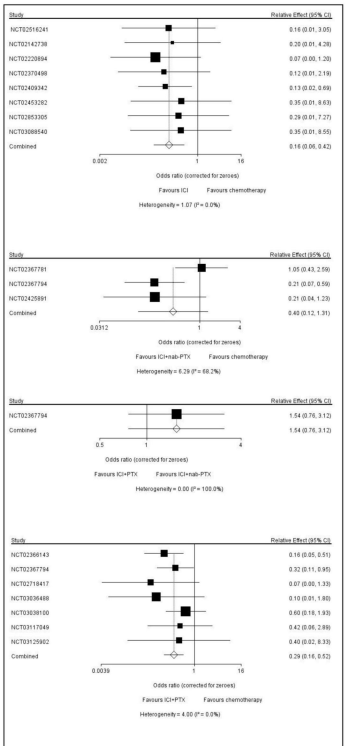

3-5 Hepatitis

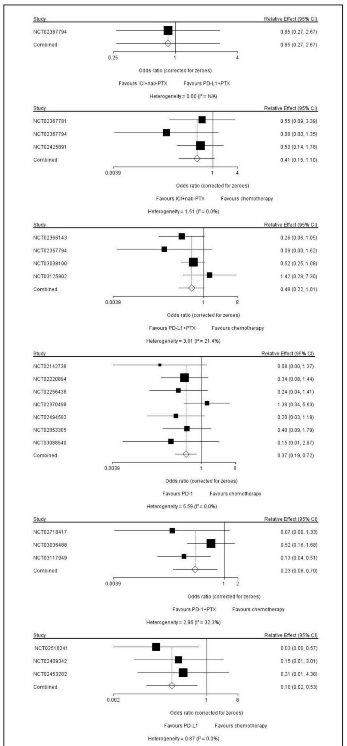

1-5 Colitis-subgroup

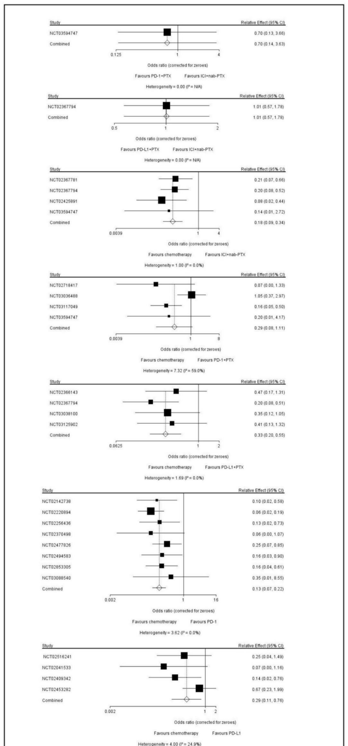

1-5 Pneumonitis-subgroup

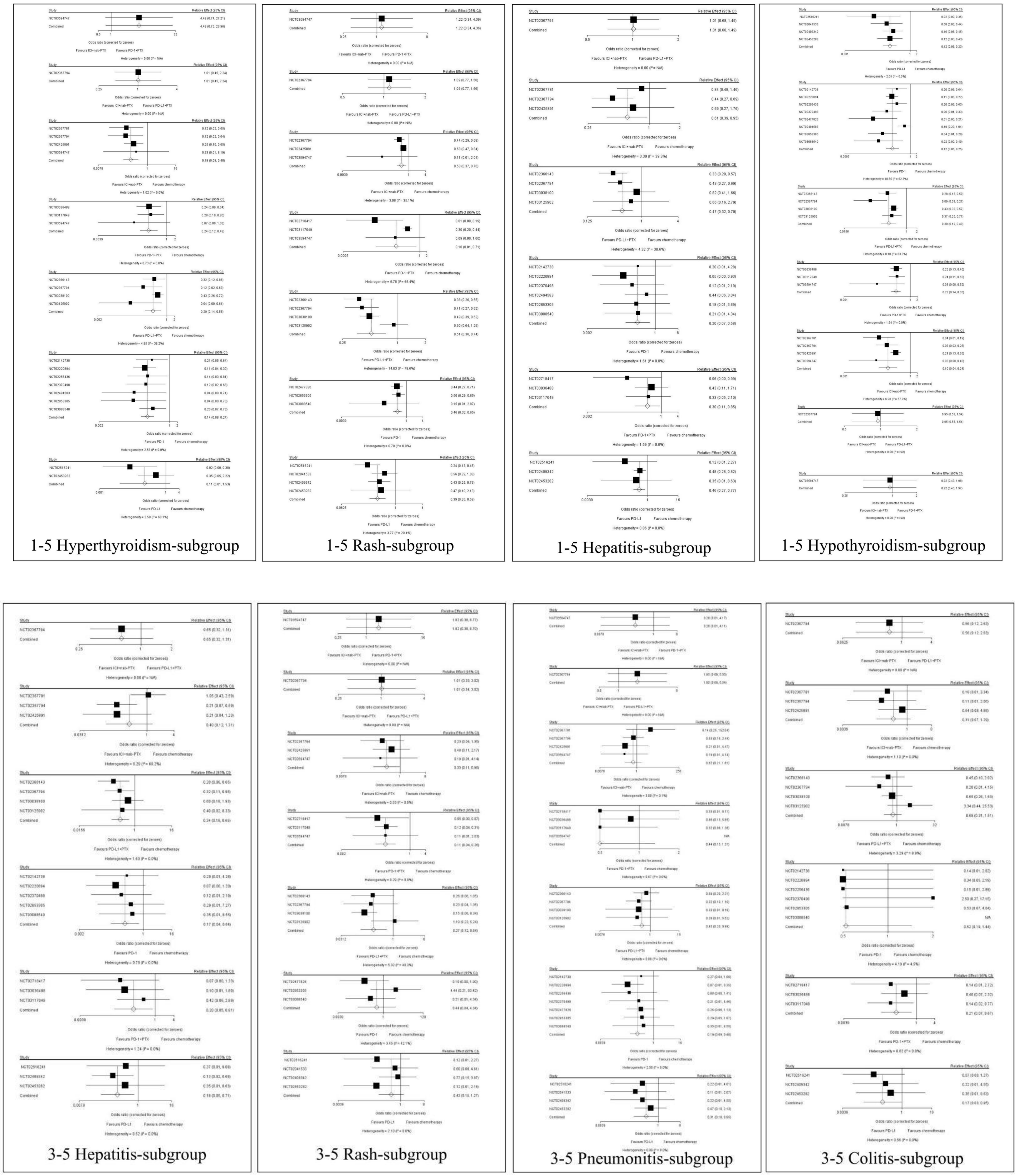

Supplementary Figure 1. Pairwise meta-analysis results for included RCTs. The results are presented as odds ratios (ORs) with corresponding 95% confidence intervals (CIs). The vertical line represents the null effect, which is set at 1. The horizontal line depicts the CIs, and the filled rectangle represents the point estimate, summarizing the ORs. When interpreting the forest plot for each pairwise comparison, it should be noted that if the filled rectangle with the entire CI does not intersect with the vertical line of null effect, a statistically significant difference is observed. If the entire CI is on the left of the null effect, the event is significantly higher

in the intervention arm, while if the entire CI (represented by a hollow diamond) is on the right, the event is statistically more frequent in the reference arm. If the entire CI intersects with the line of null effect, the difference between the two procedures is not statistically significant.

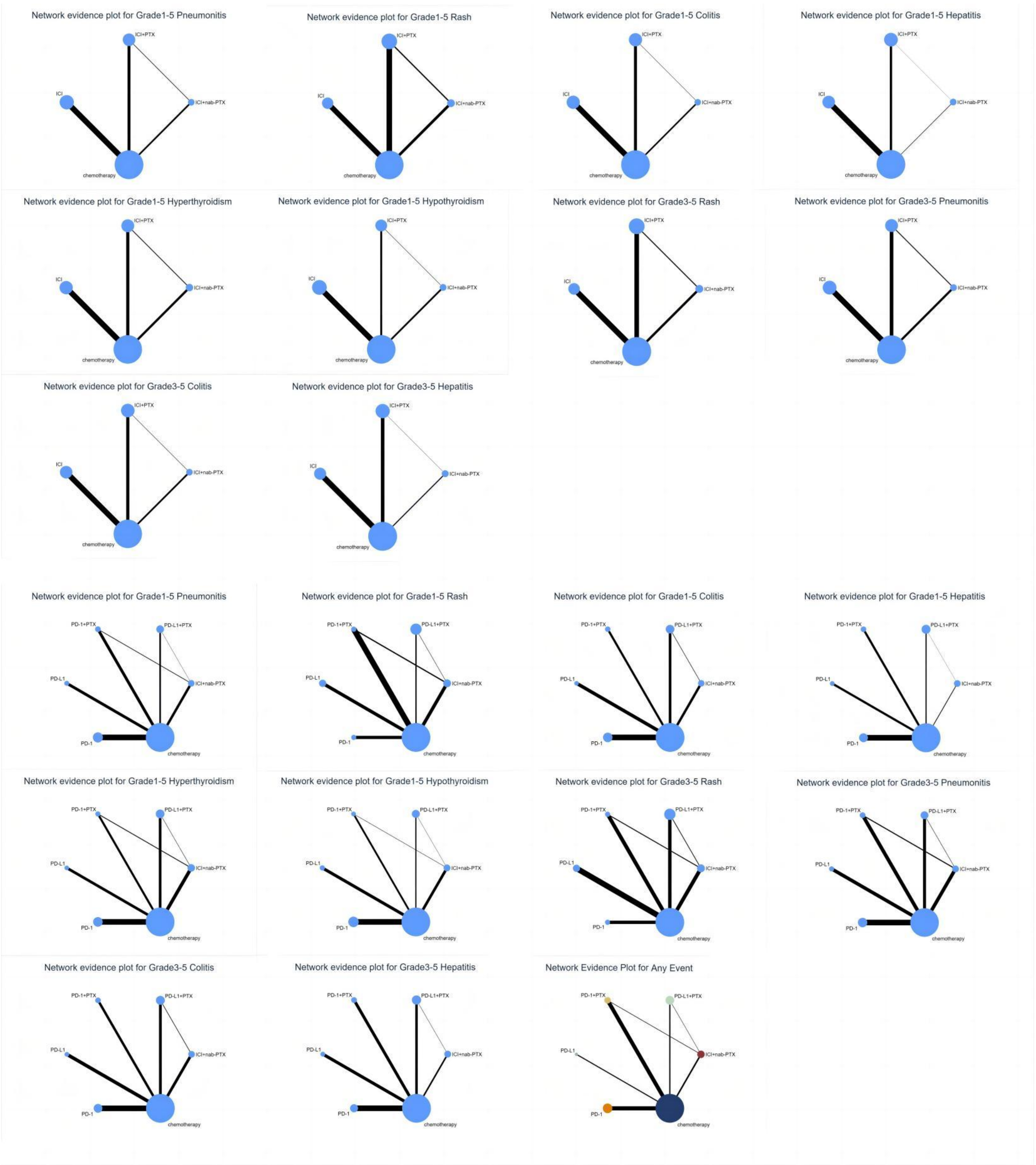

Supplementary Figure 2. Network geometry of all outcomes.The blue nodes in the figure represent the interventions being compared, while the edges represent the direct comparisons available between pairs of interventions (i.e. comparisons evaluated in at least one study). The node sizes are weighted based on the number of patients in each intervention arm, while the edges are weighted using inverse variance.

Contribution plot for Grade1-5 Any Event

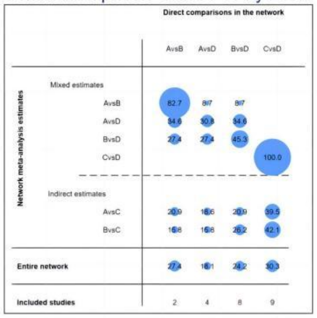

Contribution Plot for Grade3-5 Any Event

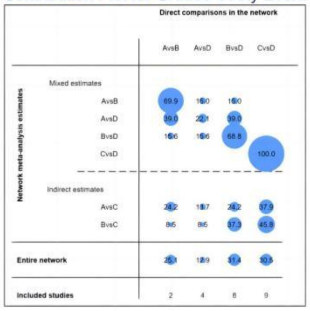

Contribution Plot for Grade1-5 Any Event

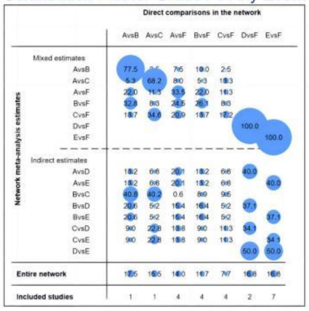

Contribution Plot for Grade3-5 Any Event

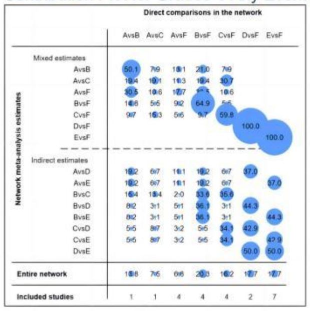

Contribution plot for Grade1-5 Pneumonitis

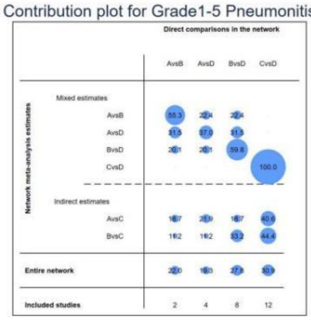

Contribution plot for Grade1-5 Rash

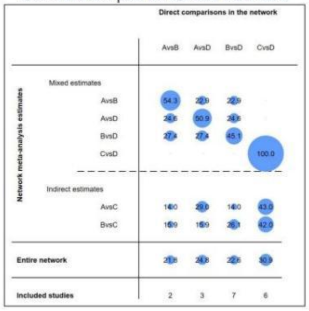

Contribution plot for Grade1-5 Colitis

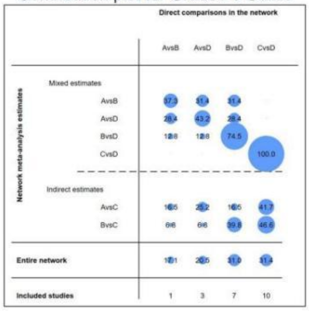

Contribution plot for Grade1-5 Hepatitis

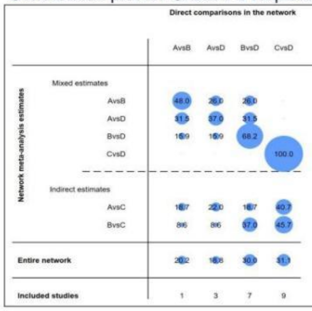

Contribution plot for Grade1-5 Hyperthyroidism

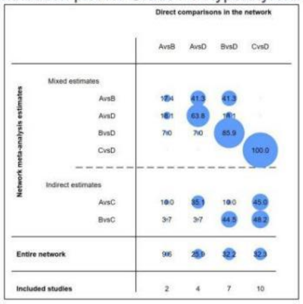

Contribution plot for Grade1-5 Hypothyroidisn

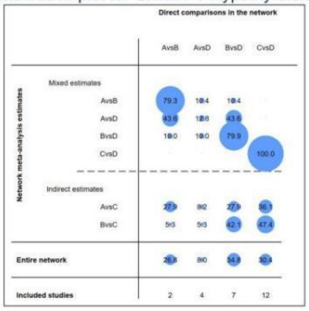

Contribution plot for Grade3-5 Rash

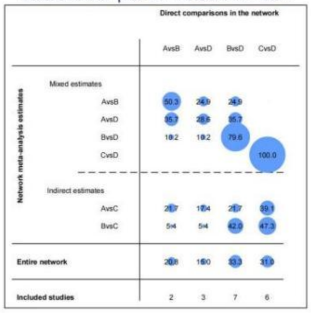

Contribution plot for (Grade3-5 neumonitis

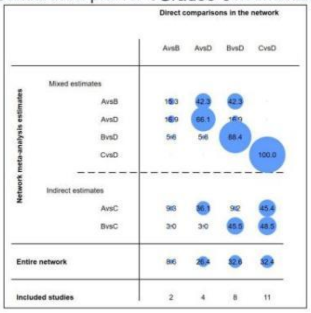

Contribution plot for Grade3-5 Colitis

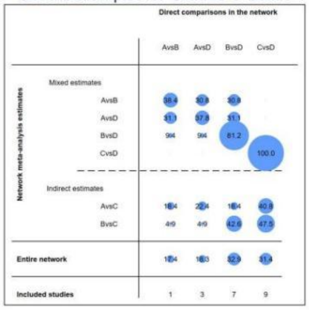

Contribution plot for Grade3-5 Hepatitis

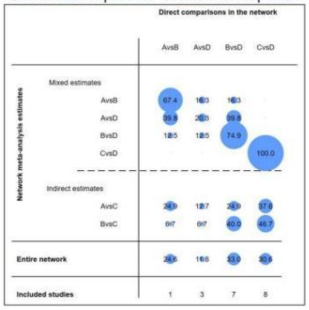

Contribution plot for Grade1-5 Rash

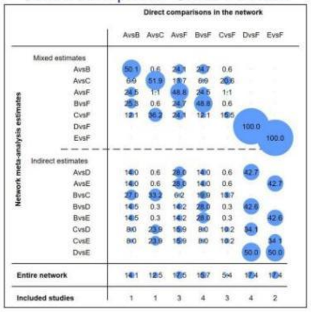

Contribution plot for Grade1-5 Pneumonitis

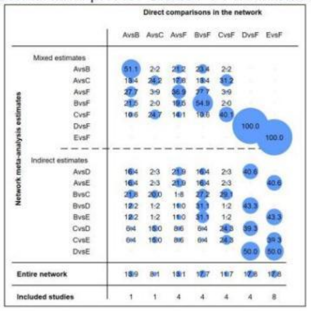

Contribution plot for Grade1-5 Hypothyroidism

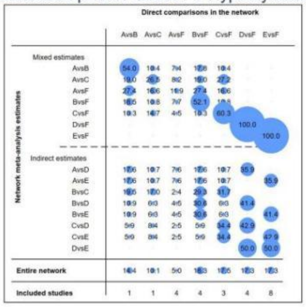

Contribution plot for Grade1-5 Colitis

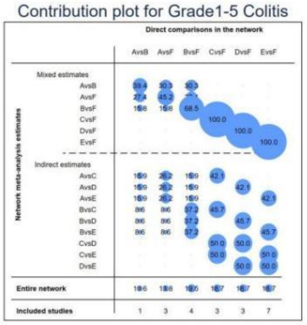

Contribution plot for Grade1-5 Hepatitis

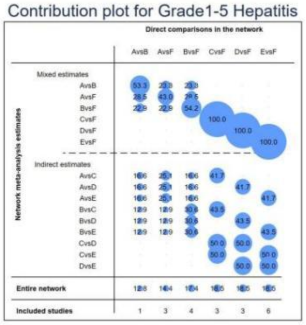

Contribution plot for Grade1-5 Hyperthyroidism

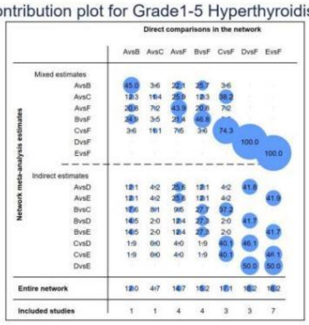

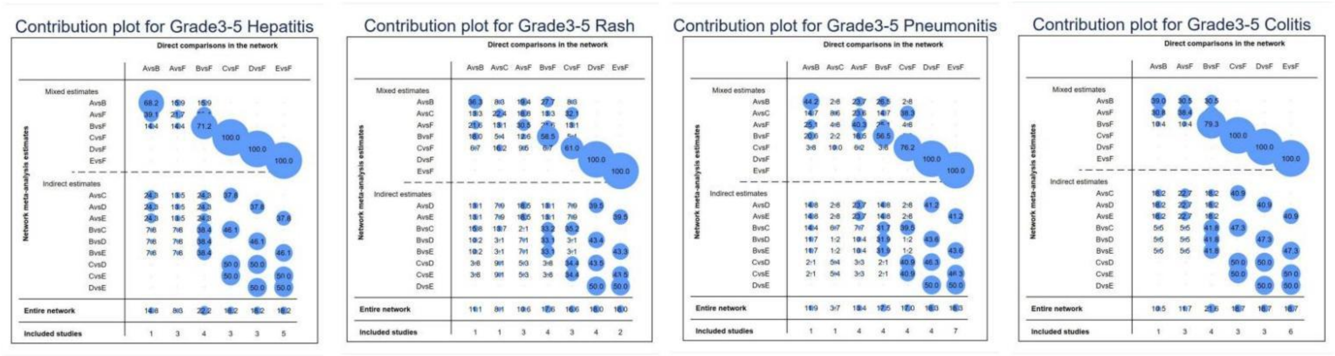

Supplementary Figure 3. Contribution plots of all outcomes. The table presents the available direct comparisons, which have been evaluated in at least one study, in its columns. The rows show the mixed comparisons (i.e., estimates already available in the literature but implemented by the network) and the indirect comparisons (i.e., comparisons not available in the literature but generated by the network). Readers should read the table from left to right, where each row displays the contribution of each direct comparison in the network estimates (mixed and indirect), and the cumulative sum of the contributions is 100%, expressed in percentages. The plots also illustrate the contribution of each direct comparison in constructing the entire network.

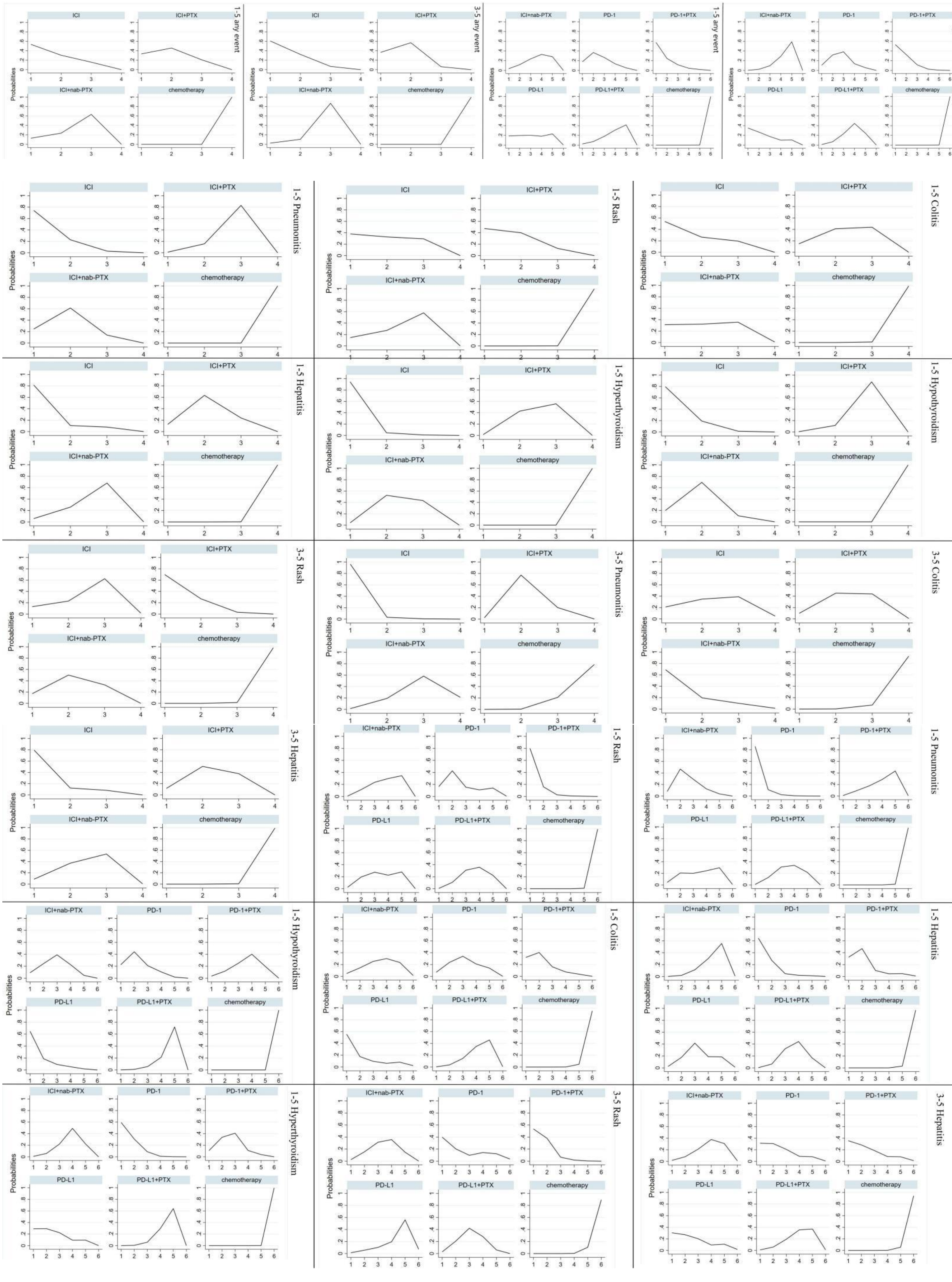

Probabilities
1-5 any event
ICI
ICI+PTX
ICI+nab-PTX
chemotherapy
3-5 any event
PD-1
PD-1+PTX
PD-L1
PD-L1+PTX
1-5 Pneumonitis
1-5 Rash
1-5 Colitis
1-5 Hepatitis
1-5 Hyperthyroidism
1-5 Hypothyroidism
3-5 Rash
3-5 Pneumonitis
3-5 Colitis
3-5 Hepatitis

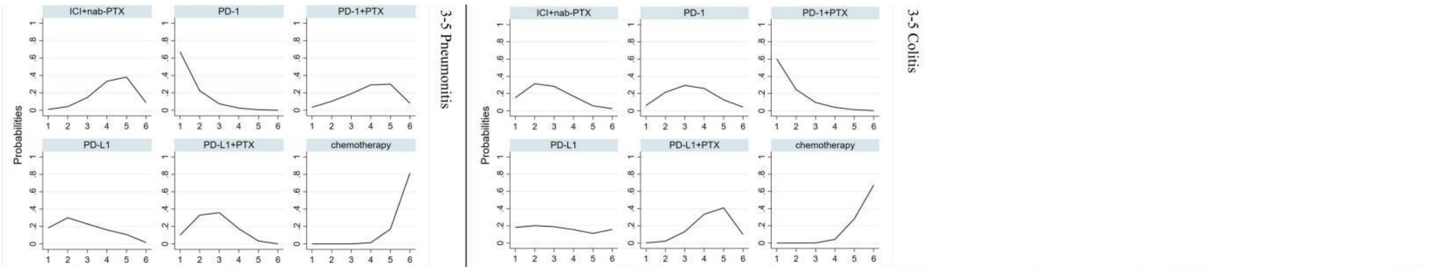

Supplementary Figure 4. Ranking profiles of treatment modalities. Ranking curves display the probability of different treatment modalities being ranked as having the highest, second-highest, third-highest, and so on, risk of immune-related adverse events.

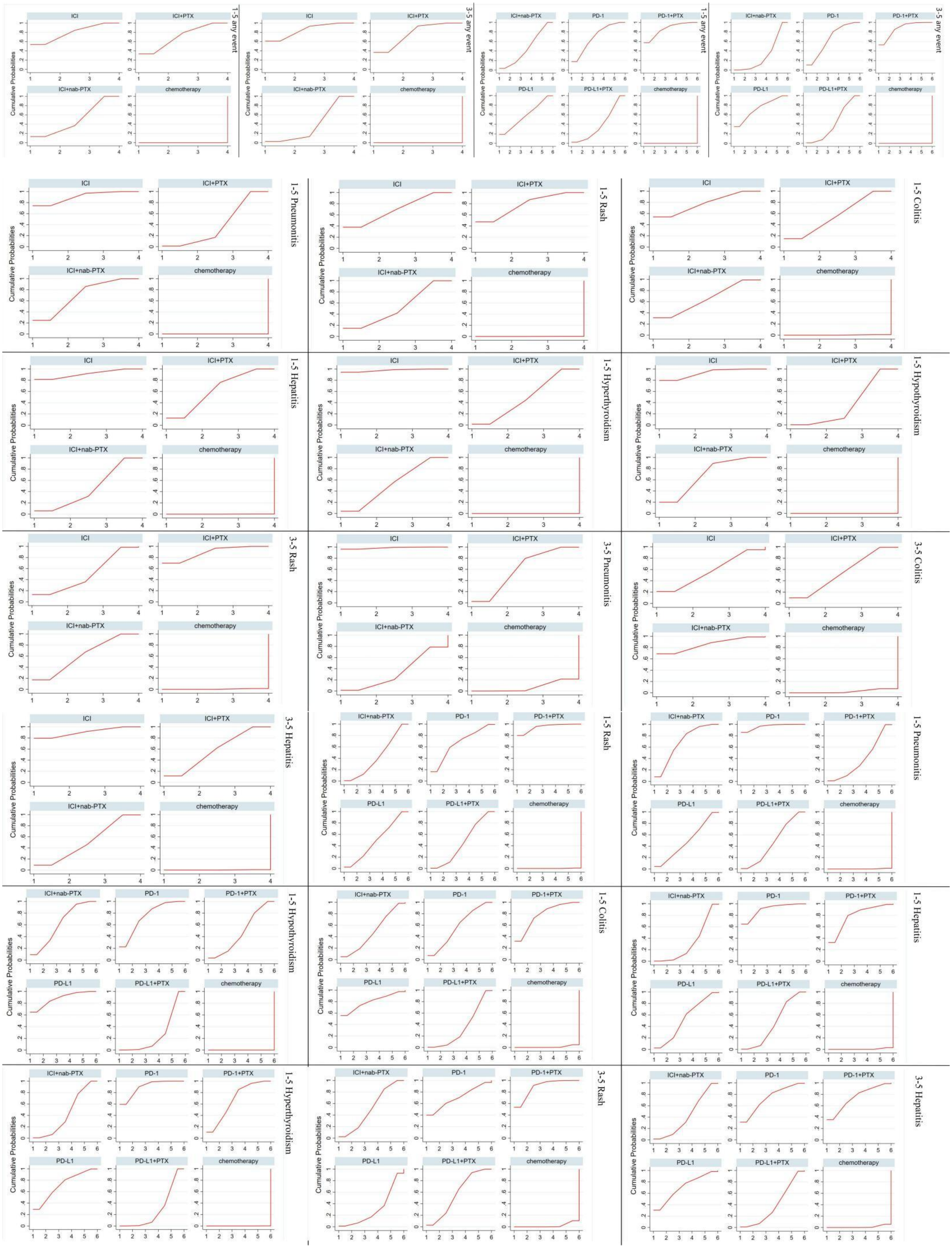

1-5 any event
3-5 any event
1-5 Pneumonitis
1-5 Rash
1-5 Colitis
1-5 Hepatitis
1-5 Hyperthyroidism
1-5 Hypothyroidism
3-5 Rash
3-5 Pneumonitis
3-5 Colitis
3-5 Hepatitis
ICI
ICI+PTX
ICI+nab-PTX
chemotherapy
PD-1
PD-1+PTX
PD-L1
PD-L1+PTX
Cumulative Probabilities

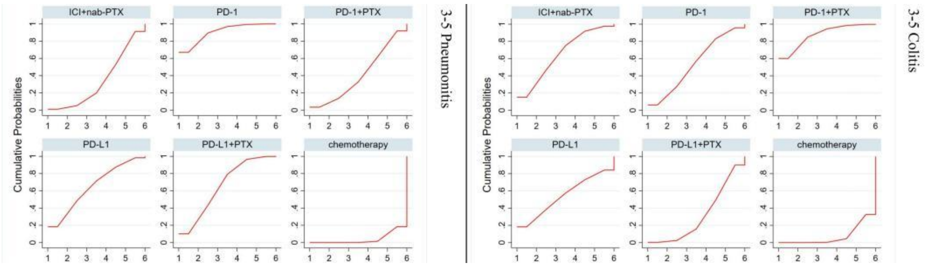

Supplementary Figure 5. The surface under the cumulative ranking (SUCRA) lines for each treatment modality. The SUCRA value ranges from 0 to 1, with higher values indicating a better rank. The SUCRA line represents the probability of each treatment being ranked at each possible position from best to worst. The area under the curve is used to calculate the SUCRA value, which can be interpreted as the percentage of the effectiveness of the treatment compared to the best possible treatment.

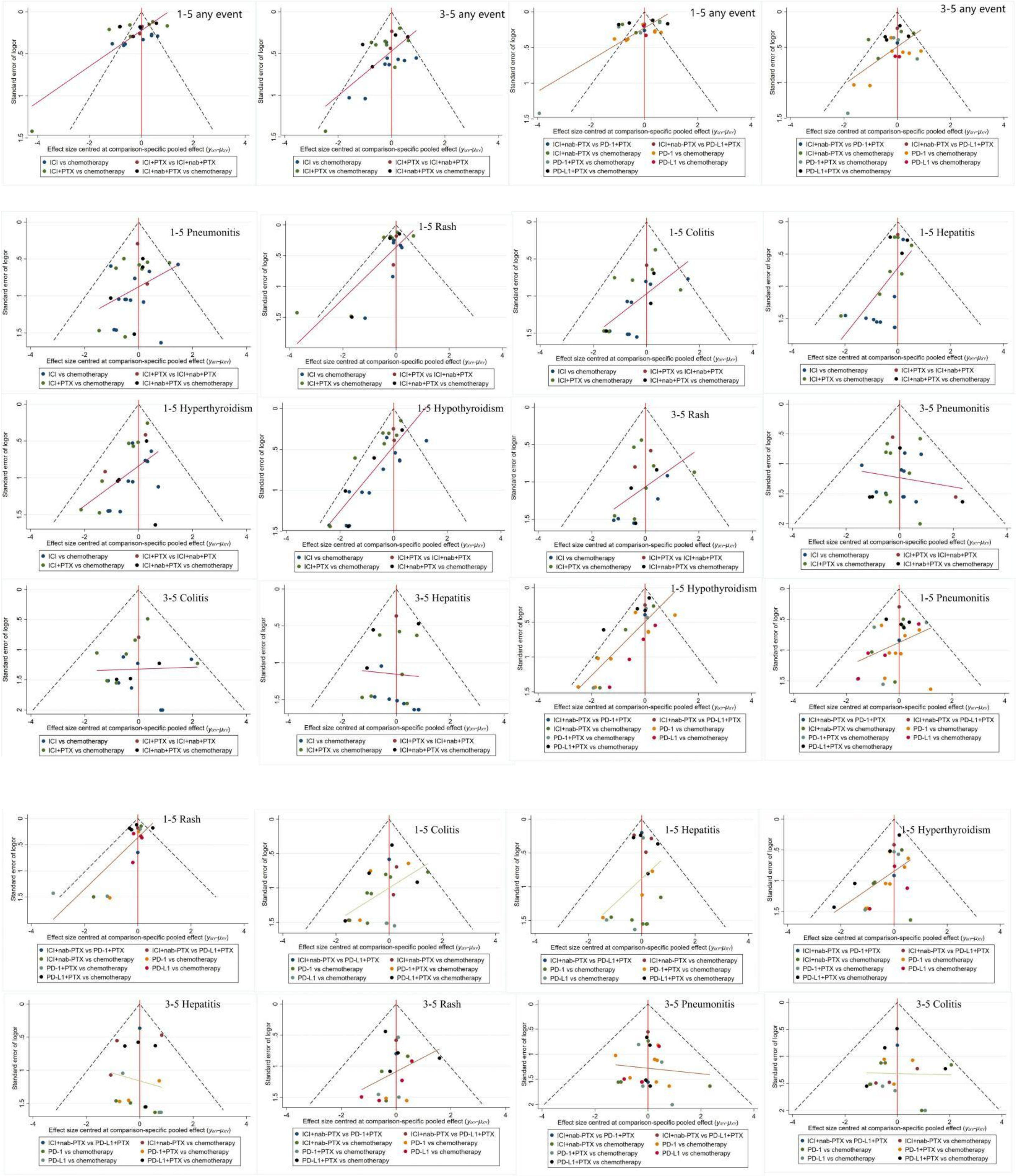

Supplementary Figure 6. Funnel plots of the network estimates of all outcomes.Funnel plots are used to assess the presence of publication bias in a systematic review. In this case, the funnel plots show the network estimates of all outcomes. In the comparison-adjusted funnel plot, the horizontal axis displays the difference between each i-study estimate YiXY and the summary effect for the respective comparison (YiXY-μXY), while the vertical axis presents the measure of dispersion of YiXY, which is the standard error of the effect size. The red

line represents the null hypothesis. Each point in the plot represents a direct comparison, with different colors representing different comparisons. The dashed black line represents the 95% confidence interval. The horizontal line represents the regression line, with the dark red regression line demonstrating the absence of asymmetry.
